# The Topological State Derivative: An Optimal Control Perspective on Topology Optimisation

**DOI:** 10.1007/s12220-023-01295-w

**Published:** 2023-05-15

**Authors:** Phillip Baumann, Idriss Mazari-Fouquer, Kevin Sturm

**Affiliations:** 1 TU Wien, Wiedner Hauptstr. 8-10, 1040 Vienna, Austria; 2 CEREMADE, UMR CNRS 7534, Université Paris-Dauphine, Université PSL, Place du Maréchal De Lattre De Tassigny, 75775 Paris cedex 16, France

**Keywords:** Topological derivative, Topology optimisation, Shape optimisation, Optimal control

## Abstract

In this paper, we introduce the topological state derivative for general topological dilatations and explore its relation to standard optimal control theory. We show that for a class of partial differential equations, the shape-dependent state variable can be differentiated with respect to the topology, thus leading to a linearised system resembling those occurring in standard optimal control problems. However, a lot of care has to be taken when handling the regularity of the solutions of this linearised system. In fact, we should expect different notions of (very) weak solutions, depending on whether the main part of the operator or its lower order terms are being perturbed. We also study the relationship with the topological state derivative, usually obtained through classical topological expansions involving boundary layer correctors. A feature of the topological state derivative is that it can either be derived via Stampacchia-type regularity estimates or alternately with classical asymptotic expansions. It should be noted that our approach is flexible enough to cover more than the usual case of point perturbations of the domain. In particular, and in the line of (Delfour in SIAM J Control Optim 60(1):22–47, 2022; J Convex Anal 25(3):957–982, 2018), we deal with more general dilatations of shapes, thereby yielding topological derivatives with respect to curves, surfaces or hypersurfaces. To draw the connection to usual topological derivatives, which are typically expressed with an adjoint equation, we show how usual first-order topological derivatives of shape functionals can be easily computed using the topological state derivative.

## Introduction

### Scope of the Paper

The main goal in shape optimisation problems is to optimise a certain set, the “design variable” ${\Omega }$, in order to maximise or minimise a certain functional. To achieve this goal, it is necessary to understand how this functional varies under perturbations of ${\Omega }$. Of particular importance are perturbations obtained by drilling a small inclusion $\omega _{\varepsilon }$ of size ${\varepsilon }$ into ${\Omega }$. The first order variation of the functional under this perturbation is called the “topological derivative”. After its introduction in the pioneering works [1–4] in the context of linear elasticity, the topological derivative framework was used in several numerical algorithms; let us for instance mention level-set algorithms [3] or Newton-type algorithm [5, Chapter 10]. We also refer to the monographs [6], where several topological derivatives for various model problems are derived.

Recently [7] a Lagrangian technique, called the “averaged adjoint approach”, was proposed as an efficient tool to compute topological derivatives. This technique allows for a wide range of applications: topological derivatives for Dirichlet boundary conditions [8], topological derivatives for nonlinear [9] and quasilinear problems [10] or higher order topological derivatives [11] can be computed in a systematic way. We also refer to [12] for another Lagrangian technique to compute topological derivatives.

In the even more recent paper [1], a way to compute the topological derivative directly using the unperturbed adjoint equation was proposed. In this reference, more general topological perturbations, called dilatations, are also considered; this leads to a more general notion of topological derivative. In [1, Thm. 3.4], the difference of the perturbed and unperturbed state variable are divided by the volume of the perturbation, however, no analysis on the existence of this limit is provided. We will see that, for the models we consider, that the limit of the quotient divided by the volume of the perturbation for point perturbations and dilatations of hypersurfaces actually exists in a suitable function space; this leads us to a new notion of topological derivative of the state which we refer to as the *t*opological state derivative. A difference between [1] and our model problems is that in this reference homogenous Neumann boundary conditions on the inclusion boundary are imposed, while we deal with transmission problems, which can be seen as inhomogenous Neumann boundary conditions on the inclusion boundary.

Let us mention other applications of asymptotic expansions, for instance in [13], where a necessary condition for the existence of a nonsmooth domain is derived. In thi paper the boundary components touch at an tangential point and thus the domain has, for instance in dimension two, a cusp at this point. Then the interesting question is also how does the asymptotic expansion of the solution look like when the two touching components are moved by a small width ${\varepsilon }>0$ and how does the solution behave. This is examined in [14], where also the asymptotic of the solution with respect to the distance ${\varepsilon }>0$ is provided. Asymptotic expansions for a variety of shape functions can be found in [15] which includes the asymptotics of eigenvalues and energy functionals. In [16, 17] an interesting self-adjoint extensions on the union of domain and ligament of width $h>0$ is studied. The asymptotics of the solution to a Poisson problem is derived with respect to the ligament thickness *h*. The ligament problem with the strip can then be approximated with the limit problem, where the ligament collapses to a curve.

Our goal, in this paper, is to present a unique view on the topological derivative, by framing it as a usual derivative, thereby leading to a direct approach to computing topological derivatives. This is done by first perturbing the partial differential equation and then deriving a linearised equation as is usually done in optimal control theory [18–20]. This shows that the design-to-state operator is actually differentiable for certain PDE constraints, and that its derivative is described by a linearised system similar to optimal control problems. These linearised systems are usually very singular in the sense that their solutions admit low regularity. Typically, for problems where the operator is perturbed, the linear system only admits very weak solutions. Interestingly these linearised systems may involve terms which are usually obtained from the classical asymptotic analysis performed on the problem under consideration. Solutions of the linearised system for the semilinear problem will be analysed in our paper through the notions introduced by Stampacchia, while the operator perturbation of the transmission problem requires the notion of very weak solutions. We remark that in state constrained optimal control problems low regularity of the adjoint equations is also an issue and thus the technical difficulties we encounter are related to the discussion of [21], where the uniqueness of solutions to adjoint equations with mixed boundary conditions is discussed. Our approach also allows us to derive at least first order topological derivatives.

***Structure of the Paper***

Our paper is structured as follows:

1. In Sect. 1.2 we gather all the basic notions and definitions of generalised topological derivatives and the topological state derivative.
2. Sect. 1.3 contains a discussion of one of our main points, that is, the link between control derivatives, topological derivatives and the asymptotic analysis of PDEs. All the rigorous computations in this section are carried out for linear operators, and serve to illustrate our idea.
3. Sect. 2 contains our rigorous results for the analysis of semilinear elliptic equations, when perturbing lower-order terms. In Subsect. 4, we study several concrete examples using adjoint states.
4. Sect. 3 is devoted to the study of point perturbations of the operator. The analysis is distinctly different from the semilinear case discussed in Sect. 2, both from the point of view of the notion of (very) weak solutions, and from that of first order asymptotics.
5. The rest of the paper contains the proofs of our results.

### Generalised Topological Derivatives and the Topological State Derivative

***Generalised Topological Derivatives***

Throughout the paper, we let ${\textsf{D}}\subset {\textbf{R}}^d$ be a design region that is, a smooth, open, bounded domain. Henceforth we denote by ${{\mathcal {A}}}({\textsf{D}})$ the set of admissible designs; in other words,

$$\begin{aligned} {{\mathcal {A}}}({\textsf{D}})=\left\{ {\Omega }\text { measurable, }{\Omega }\subset {\textsf{D}}\right\} . \end{aligned}$$

A function $J:{{\mathcal {A}}}({\textsf{D}})\rightarrow {\textbf{R}}$ is called a shape functional.

#### Definition 1.1

Let $\Omega \in {{\mathcal {A}}}({\textsf{D}})$. Consider a compact set $E\subset {\textsf{D}}$ such that $\partial \Omega \cap E=\emptyset$ and denote by $E_{\varepsilon }:= \{x\in {\textbf{R}}^d:\; d_E(x)< {\varepsilon }\}$ the tubular neighborhood of *E* of width ${\varepsilon }>0$. We define the perturbed set $\Omega (E_{\varepsilon })\subset {\textsf{D}}$ by

1 $$\begin{aligned} {\Omega }(E_{\varepsilon }):= {\left\{ \begin{array}{ll} {\Omega }\cup E_{\varepsilon }&{} E\subset {\textsf{D}}\setminus {\overline{{\Omega }}}, \\ {\Omega }\setminus \overline{E}_{\varepsilon }&{} E\subset {\Omega }. \end{array}\right. } \end{aligned}$$

The topological derivative of the functional *J* at *E* is defined by the following limit, provided it exists:

2 $$\begin{aligned} DJ({\Omega })(E) := \lim _{{\varepsilon }\searrow 0} \frac{J({\Omega }(E_{\varepsilon })) - J({\Omega })}{|E_{\varepsilon }|}. \end{aligned}$$

#### Remark 1.2

Here, we note that our definition of topological derivative already assumes that the first order term in the asymptotic expansion of *J* is of order $|E_{\varepsilon }|$, the Lebesgue measure of $E_{\varepsilon }$. This obviously depends on the shape functional under consideration. In several cases, for instance when considering a PDE dependent shape functional, and when enforcing Dirichlet boundary conditions on the boundary of $E_{\varepsilon }$, terms of lower order appear [8]. However, as will be clear throughout, in all cases under consideration here, the leading order in the topological expansion is $|E_{\varepsilon }|$.

Working with tubular neighborhoods allows for a great variety of perturbations; let us list a few examples corresponding to particular choices of *E*.

#### Examples 1.3

Assume again that ${\textsf{D}}\subset {\textbf{R}}^d$ and ${\Omega }\subset {\textsf{D}}$.

- $E=\{x_0\}$, $x_0\in {\textsf{D}}$. Then $E_{\varepsilon }= B_{\varepsilon }(x_0)$ and $|E_{\varepsilon }| = {\varepsilon }^d|B_1(0)|$, where $B_r(x)$ denotes the open ball of radius $r>0$ located at *x* in ${\textbf{R}}^d$.
- Let $E=\Gamma \subset {\textsf{D}}$ be a smooth closed orientable hypersurface with normal $\nu :\Gamma \rightarrow {\textbf{R}}^d$, $|\nu |=1$ on $\Gamma$. Then, for ${\varepsilon }>0$ small enough, $\Gamma _{\varepsilon }= \{x+t\nu (x):\; x\in \Gamma :\; t\in [0,{\varepsilon })\}$ and $|\Gamma _{\varepsilon }| = {\varepsilon }\textrm{Per}(\Gamma )+o_{{\varepsilon }\rightarrow 0}({\varepsilon }),$ where $\textrm{Per}(\Gamma )$ the perimeter of $\Gamma$, which in view of the smoothness of $\Gamma$ is equal to the $(d-1)$-dimensional Lebesgue measure of $\Gamma$.

***The Topological State Derivative as Derivative of the Shape-to-State Operator***

Throughout this paper, we only consider PDE-dependent shape functionals. Let $X({\textsf{D}})$ be a space of functions defined on ${\textsf{D}}$ with values in ${\textbf{R}}$. We consider an equation of the type: find $u_{\Omega }\in X({\textsf{D}})$, such that

3 $$\begin{aligned} \langle E_{\Omega }(u_{\Omega }),\varphi \rangle _{X({\textsf{D}})',X({\textsf{D}})}=0 \quad \text { for all } \varphi \in X({\textsf{D}}), \end{aligned}$$

where $E_{\Omega }:X({\textsf{D}})\rightarrow X({\textsf{D}})'$ is a potentially nonlinear operator. Typically, $X({\textsf{D}})$ is a Sobolev space ($X({\textsf{D}})=W^{1,p}({\textsf{D}})$), and (3) merely corresponds to the weak formulation of an elliptic equation of the type

4 $$\begin{aligned} {\left\{ \begin{array}{ll} {\mathcal {L}}_{\Omega }u=f_{\Omega }\quad \text { in }{\textsf{D}}\,, \\ u\text { satisfies boundary conditions on }\partial {\textsf{D}}, \end{array}\right. } \end{aligned}$$

where the expression “weak formulation” needs to be specified. The operator ${\mathcal {L}}_{\Omega }$ depends on ${\Omega }$. In this paper, several dependences on ${\Omega }$ are considered: ${\mathcal {L}}_{\Omega }$ can take the form $-{\text {div}}((\alpha +\beta \chi _{\Omega })\nabla )$, or $-\Delta -\chi _{\Omega }$, and can be nonlinear in *u*. Similarly, the function $f_{\Omega }$ is *a priori* assumed to depend on the set ${\Omega }$.

#### Definition 1.4

We define the shape-to-state operator $S:{{\mathcal {A}}}({\textsf{D}}) \rightarrow X({\textsf{D}})$ by $S({\Omega }):= u_{\Omega }$, where $u_{\Omega }$ solves (3) for the set ${\Omega }\in {{\mathcal {A}}}({\textsf{D}})$.

Of course, under proper assumptions on the nonlinear operator $E_{\Omega }$, *S* is a uniquely defined operator so that Definition 1.4 makes sense.

In the following definition, we introduce the shape-to-state operator and its derivative, which we refer to as the topological state derivative. In contrast to the usual asymptotic expansion [6, Chapter 5] of the state, our definition does not involve a rescaling and is simply the usual differential quotient of the state; in this regard, it is akin to an optimal control approach.

#### Definition 1.5

(*Topological state derivative: derivative of the shape-to-state operator*) Let ${\Omega }\in {{\mathcal {A}}}({\textsf{D}})$ and consider a compact set $E\subset {\textsf{D}}{\setminus }{\overline{{\Omega }}}$ or $E\subset {\Omega }$. For ${\varepsilon }>0$ we introduce

5 $$\begin{aligned} U_{\varepsilon }:= U_{{\Omega }(E_{\varepsilon })} := \frac{u_{{\Omega }(E_{\varepsilon })} - u_{{\Omega }}}{|E_{\varepsilon }|}, \end{aligned}$$

and define the *topological state derivative* of *S* at ${\Omega }$ in direction *E* by

6 $$\begin{aligned} S'({\Omega })(E) := U_0:= U_{E,0} := \lim _{{\varepsilon }\searrow 0} U_{\varepsilon }, \end{aligned}$$

where the limit has to be understood in an appropriate function space specified later on.

In the following sections we will examine three different PDE constraints and study the differentiability of the corresponding shape-to-state operator. This will form the groundwork for the optimisation of several PDE constrained functionals.

### Control Derivatives, Topological Derivatives and Asymptotic Analysis

***From Control Derivatives to Topological Derivatives***

When using the wording “control derivative”, what we mean is that the shape ${\Omega }\in {{\mathcal {A}}}({\textsf{D}})$ is identified with its characteristic function $\chi _{\Omega }$, and that we actually consider variations of ${\Omega }$ as variations of $\chi _{\Omega }$. To give this concept a more precise meaning, let us take a basic example: for every ${\Omega }\in {{\mathcal {A}}}$, let $u_{\Omega }\in H^{1}({\textsf{D}})$ be the unique solution of

7 $$\begin{aligned} \left\{ \begin{alignedat}{2} -\Delta u_{\Omega }&=\chi _{\Omega }\quad{} & {} \text { in }{\textsf{D}},\\ u_{\Omega }&=0 \quad{} & {} \text { on }\partial {\textsf{D}}. \end{alignedat}\right. \end{aligned}$$

Let $E\subset {\textsf{D}}$ be either a point or a smooth oriented hypersurface, and assume for the sake of simplicity that $E\subset {\textsf{D}}\backslash \overline{{\Omega }}$. Then, for ${\varepsilon }>0$ small enough, we have, with $\Omega _{\varepsilon }:= \Omega (E_{\varepsilon })= \Omega \cup E_{\varepsilon }$,

$$\begin{aligned} \chi _{{\Omega }_{\varepsilon }}=\chi _{{\Omega }}+\chi _{E_{\varepsilon }}, \end{aligned}$$

so that, setting $\mu _{\varepsilon }=\frac{\chi _{E_{\varepsilon }}}{|E_{\varepsilon }|}$, the function $U_{\varepsilon }:=\frac{u_{{\Omega }_{\varepsilon }}-u_{\Omega }}{|E_{\varepsilon }|}$ solves

8 $$\begin{aligned} \left\{ \begin{alignedat}{2} -\Delta U_{\varepsilon }&=\mu _{\varepsilon }{} & {} \quad \text { in }{\textsf{D}},\\ U_{\varepsilon }&=0{} & {} \quad \text { on } \partial {\textsf{D}}. \end{alignedat}\right. \end{aligned}$$

For each ${\varepsilon }>0$ the function $\mu _{\varepsilon }$ is a probability measure on ${\textsf{D}}$.

In the case where $E=\{x_0\}$, it is clear that $\mu _{\varepsilon }\rightharpoonup \delta _{x_0}$ as ${\varepsilon }\searrow 0$ weakly in the sense of measures and it is then expected that $\{U_{\varepsilon }\}_{{\varepsilon }>0}$ converges in some sense to the solution $U_{\{x_0\},0}\in X({\textsf{D}})$ of the elliptic equation (with measure datum)

9 $$\begin{aligned} \left\{ \begin{alignedat}{2} -\Delta U_{\{x_0\},0}&=\delta _{x_0}{} & {} \quad \text { in }{\textsf{D}},\\ U_{\{x_0\},0}&=0{} & {} \quad \text { on }\partial {\textsf{D}}. \end{alignedat}\right. \end{aligned}$$

To make the function space $X({\textsf{D}})$ precise, we will require some background information on the weak formulation of (9), but what matters is that the topological state derivative appears, in this case, as the Green kernel of $-\Delta$. This simple remark allows to go back from topological derivatives to control derivatives.

***Expressing Control Derivatives Via the Topological State Derivative***

Indeed, assume we wish to compute the control derivative of (8); this means that we see $\chi _{\Omega }$ as a function in $L^2({\textsf{D}})$ and that we consider the control derivative of the state, defined, for a given perturbation *h*, as

$$\begin{aligned} \dot{u}_h:=\lim _{t \searrow 0}\frac{v_{t h}-u_{\Omega }}{t} \end{aligned}$$

where $v_{t h}\in L^2({\textsf{D}})$ satisfies (8) with $\chi _{\Omega }$ replaced with $\chi _{\Omega }+t h$. Then it is clear, by linearity of the equation, that $\dot{u}_h$ satisfies

10 $$\begin{aligned} \left\{ \begin{alignedat}{2} -\Delta \dot{u}_h&=h{} & {} \quad \text { in }{\textsf{D}}, \\ \dot{u}_h&=0{} & {} \quad \text { on }\partial {\textsf{D}}. \end{alignedat}\right. \end{aligned}$$

As we already explained briefly that the topological state derivative coincides with the Green kernel of the operator $-\Delta$, it is reasonable to expect, for instance if *h* is supported in ${\textsf{D}}\backslash {\overline{{\Omega }}}$, that $\dot{u}_h$ writes as

$$\begin{aligned} \dot{u}_h(x)=\int _{{\textsf{D}}\backslash {\Omega }} h(y)U_{\{y\},0}(x)dy. \end{aligned}$$

Consequently, we see on this simple example that the knowledge of the topological derivative implies that we are able to compute any control-type derivative. One of our objectives in this paper is to prove the validity of this intuitive paradigm in several cases.

Of course, several points need to be underlined here. First and foremost, as should be clear, we need to work with elliptic equations with measure data in order to obtain optimal estimates. Most of this will be done using and adapting the techniques of [22], which itself relies on the seminal [23]. Second, a lot of care needs to be taken when differentiating nonlinear problems, and giving proper regularity estimates on the fundamental solutions of the linearised operator; here, we rely on the aforementioned [23]. Finally, as we shall see, the weak formulation of the equation on $U_{\{x_0\},0}$ will be strongly dependent on the type of perturbation we consider. While, for perturbation of lower-order terms, the setting correspond to the standard one, we need to introduce a notion of very weak solution when considering transmission-type problems.

***Asymptotics of the Shape-to-State Operator of Point Perturbations***

Our goal is now to link the control derivatives and the usual asymptotic analysis of the shape-to-state operator.

“Singular” perturbations (i.e. removing a ball in the domain) and the asymptotics of PDEs where the singular perturbation appears are usually treated by introducing so-called “boundary layer correctors”. This approach typically involves working in unbounded domains. Although working with an optimal control approach allows to only work in bounded domains, this limit layer approach is of great importance in topology optimisation and we thus present it in this paragraph. We refer to [24, 25] for the asymptotic analysis of such singular perturbations and to [5, 6] for computations of topological derivatives of shape functionals. In contrast to these more classical approaches, we recall in this section the point of view of [9, 11, 26], which, while also using boundary-layer correctors, rescales the domain to keep a fixed size of the inclusion. As shown in [9, 10], this approach can be advantageous when dealing with semilinear and quasilinear PDEs. For this reason we give the following definition:

#### Definition 1.6

(*Derivative of shape-to-state operator, the rescaled domain approach*) Let $x_0\in {\textsf{D}}$. Define $E:=\{x_0\}$ and consider a connected and bounded domain $\omega \subset {\textbf{R}}^d$ with $0\in \omega$. For any ${\varepsilon }>0$, we define the diffeomorphism $T_{\varepsilon }:{\textsf{D}}\ni x\mapsto x_0+{\varepsilon }x$ and the rescaled domain

$$\begin{aligned}{\textsf{D}}_{\varepsilon }:=T_{\varepsilon }^{-1}({\textsf{D}}).\end{aligned}$$

Introduce $\omega _{\varepsilon }(x_0):= x_0 + {\varepsilon }\omega$ and define

11 $$\begin{aligned} \Omega _{\varepsilon }(x_0,\omega ) := {\left\{ \begin{array}{ll} \Omega \cup \omega _{\varepsilon }(x_0) &{} \text { for }x_0\in {\textsf{D}}\setminus {\overline{\Omega }}, \\ \Omega \setminus \overline{\omega _{\varepsilon }(x_0)} &{} \text { for } x_0 \in \Omega . \end{array}\right. } \end{aligned}$$

Note that according to Definition 1.1, we have ${\Omega }_{\varepsilon }(x_0,\omega )={\Omega }(\omega _{\varepsilon }(x_0))$. However, we introduce the notation ${\Omega }_{\varepsilon }(x_0,\omega )$ to emphasise the dependence on both $x_0$ and $\omega$. Furthermore, we set $u_{\varepsilon }:= u_{{\Omega }_{\varepsilon }(x_0,\omega )}$, $u_0:= u_{\Omega }$ and finally define

$$\begin{aligned} K_{\varepsilon }:=\frac{(u_{\varepsilon }- u_0)\circ T_{\varepsilon }}{{\varepsilon }}, \quad {\varepsilon }>0. \end{aligned}$$

The derivative of the shape-to-state operator is

12 $$\begin{aligned} K:= \lim _{{\varepsilon }\searrow 0} K_{\varepsilon }, \end{aligned}$$

where the limit has to be understood in an appropriate setting. We note that the limit *K* typically depends on $x_0$, $\omega$ and as well as $\Omega$. As the domain of definition ${\textsf{D}}_{\varepsilon }$ of $K_{\varepsilon }$ varies with ${\varepsilon }$, (12) needs to be understood as $\Vert K_{\varepsilon }-K\Vert _{X({\textsf{D}}_{\varepsilon })}\rightarrow 0$ for the norm of a suitable function space $X({\textsf{D}}_{\varepsilon })$.

The function *K* typically satisfies an equation in an unbounded domain. We refer to the later sections for examples and also to [9, 11, 26, 27] for concrete topological derivative examples using the rescaling approach outlined above.

#### Remark 1.7

Let us underline that this definition covers the case of ball perturbations which corresponds to $\omega =B_1(0)$ in the previous definition, and is more general in the sense that shapes other than a ball are allowed. However, it does not include lower-dimensional objects. This is in contrast with Definition 1.5.

***Connection Between Asymptotics of State and Topological State Derivative***

We consider again the problem of the previous section, namely,

13 $$\begin{aligned} \left\{ \begin{alignedat}{2} -\Delta u_{\Omega }&=\chi _{\Omega }{} & {} \quad \text { in }{\textsf{D}},\\ u_{\Omega }&=0{} & {} \quad \text { on }\partial {\textsf{D}}. \end{alignedat}\right. \end{aligned}$$

We now sketch the connection between the asymptotic expansion of $$u_{\Omega _{\varepsilon }(x_0,\omega )}$$ for $$x_0\in {\textsf{D}}{\setminus }\overline{\Omega }$$ and the topological state derivative. So we restrict ourselves to point perturbations and note that $$\Omega _{\varepsilon }(x_0,\omega ) = \Omega \cup \{x_0 + {\varepsilon }\omega \}$$. We only discuss the case $$d=3$$ and provide the results for $$d=2$$ in later sections. In the fixed three dimensional domain $${\textsf{D}}$$, with $$f_1=1, f_2=0 $$, we have [26] the following expansion of $$u_{\Omega _{\varepsilon }(x_0,\omega )}=:u_{\varepsilon }$$:

14 $$\begin{aligned} u_{\varepsilon }(x) = u_0(x) + {\varepsilon }^2(K(T_{\varepsilon }^{-1}(x)) + {\varepsilon }v(x)) + \text { higher order terms}, \quad \text { for a.e. } x\in {\textsf{D}}, \end{aligned}$$

where $u_0:=u_\Omega$ and *v* is a regular boundary corrector function defined on the fixed domain ${\textsf{D}}$. Then in fact we will show that almost everywhere one can indeed recover the topological state derivative via the limit

15 $$\begin{aligned} U_0(x) = \lim _{{\varepsilon }\searrow 0} \frac{u_{\varepsilon }-u_0}{|\omega _{\varepsilon }|} = \lim _{{\varepsilon }\searrow 0} \frac{1}{|\omega _{\varepsilon }|}{\varepsilon }^2(K(T_{\varepsilon }^{-1}(x)) + {\varepsilon }v(x)), \quad x\in {\textsf{D}}. \end{aligned}$$

The function *K* admits the asymptotic behaviour $K(x) = R(x) + O(|x|^{-2})$ with $R(x):= |\omega | E(x)$ and $E(\cdot )$ being the fundamental solution of $-\Delta$ on ${\textbf{R}}^3$:

16 $$\begin{aligned} E(x) = \frac{1}{4\pi |x|}, \end{aligned}$$

where here and henceforth we denote by |*x*| the Euclidean norm of a vector $x\in {\textbf{R}}^d$. From the first asymptotic term of *K*, which is *R*, we can also determine the corrector $v\in H^1({\textsf{D}})$ as the solution of

17 $$\begin{aligned} \left\{ \begin{alignedat}{2} -\Delta v&=0{} & {} \quad \text { in } {\textsf{D}}\,, \\ v(x)&= -R(x-x_0){} & {} \quad \text { on }\partial {\textsf{D}}. \end{alignedat}\right. \end{aligned}$$

Therefore, one can compute the first limit on the right hand side of (15) explicitly using $R(T_{\varepsilon }^{-1}(x)) = {\varepsilon }R(x-x_0)$:

$$\begin{aligned} \lim _{{\varepsilon }\searrow 0} \frac{1}{|\omega _{\varepsilon }|}{\varepsilon }^2(K(T_{\varepsilon }^{-1}(x))&= \lim _{{\varepsilon }\searrow 0} \frac{1}{|\omega _{\varepsilon }|}{\varepsilon }^2 R(T_{\varepsilon }^{-1}(x)) \\&= |\omega |^{-1}R(x-x_0) = \frac{1}{ 4\pi |x-x_0|}. \end{aligned}$$

We note that $x\mapsto R(x-x_0)\in W^{1,q}({\textsf{D}})$ for $q\in [1,\frac{d}{d-1}) = [1,\frac{3}{2})$. Summarising, we derived the following form of $U_0$:

18 $$\begin{aligned} U_0(x) = |\omega |^{-1}( R(x-x_0) + v(x)) \quad \text { for a.e. } x\in {\textsf{D}}, \end{aligned}$$

and thus conclude that $U_0$ is indeed a solution of

19 $$\begin{aligned} \left\{ \begin{alignedat}{2} -\Delta U_0&=\delta _{x_0}{} & {} \quad \text { in }{\textsf{D}}, \\ U_0&=0{} & {} \quad \text { on } \partial {\textsf{D}}. \end{alignedat}\right. \end{aligned}$$

The solution $|\omega |^{-1}\left( R(x-x_0)+v(x)\right)$ is a well-known splitting for (19) and is often used in numerical analysis [28, 29]. The function $|\omega |^{-1}R(x-x_0)$ solves the Poisson equation in ${\textbf{R}}^3$ with Dirac measure at $x_0$ as a right hand side, and $|\omega |^{-1}v(x)$ corrects the boundary error introduced by $|\omega |^{-1}R(x-x_0)$, so that $U_0$ has homogeneous Dirichlet boundary conditions on $\partial {\textsf{D}}$.

In conclusion, the topological state derivative can be obtained from the asymptotic analysis of the state equation. If the asymptotic analysis of the state equation is performed using compound asymptotics [6, 24, 25], then one naturally obtains a splitting for the limit solution into a regular part, which comes from the corrector *v* and an irregular part, which originates from the corrector *K*. We will see later that the topological expansion can be effectively used to compute the topological state derivative and even establish strong convergence in suitable function spaces.

## Main Results for General Topological Perturbations of Semilinear Equations

We first give some basic results about the convergence in measure of the functions $\chi _{E_{\varepsilon }}$ (see Definition 1.1 for the definition of $E_{\varepsilon }$). In the following sections, we proceed with steps of increasing complexity, first considering topological state derivatives for lower order terms, then considering transmission problems.

### Convergence in Measure of $\chi _{E_{\varepsilon }}$ and Notation

Our goal is to make sense of topological derivatives for any type of *d*-dimensional inclusions as proposed in [2]. For this reason, we need to specify the behaviour of $\chi _{E_{\varepsilon }}$, as ${\varepsilon }\rightarrow 0$. This is the object of the following proposition; it is stated without a proof as it is fairly standard.

#### Proposition 2.1

For every nonempty compact $E\subset {\textsf{D}}$ and for every ${\varepsilon }>0$ we let $E_{\varepsilon }$ be its tubular neighborhood (see Definition 1.1) and we consider the probability measure on ${\textsf{D}}$

$$\begin{aligned} \mu _{E_{\varepsilon }}:=\frac{\chi _{E_{\varepsilon }}}{|E_{\varepsilon }|}. \end{aligned}$$

1. Assume $E=\{x_0\}$, so that $E_{\varepsilon }=B_{\varepsilon }(x_0)$. Then, for the weak convergence of measures,
  $$\begin{aligned} \mu _{E_{\varepsilon }}\underset{{\varepsilon }\searrow 0}{\rightharpoonup }\mu _{E}:=\delta _{x_0}. \end{aligned}$$
2. Assume $E=\Gamma$ is a $(d-1)$-dimensional Lipschitz hypersurface with finite perimeter $\textrm{Per}(\Gamma )$. Then, in the sense of measures,
  $$\begin{aligned} \mu _{E_{\varepsilon }}\underset{{\varepsilon }\searrow 0}{\rightharpoonup }\mu _{E}:=\frac{1}{\textrm{Per}(\Gamma )} ({{\mathcal {H}}}^{d-1}\lfloor \Gamma ), \end{aligned}$$
  where ${{\mathcal {H}}}^{d-1}\lfloor \Gamma$ stands for the restriction of the $(d-1)$-dimensional Hausdorff measure to $\Gamma$.
3. Assume that $E=M$ is a $1< k < d-1$ dimensional compact and smooth submanifold (without boundary). Then, in the sense of measures:
  $$\begin{aligned} \mu _{E_{\varepsilon }}\underset{{\varepsilon }\searrow 0}{\rightharpoonup }\mu _{E}:=\frac{1}{{{\mathcal {H}}}^k(M)} ({{\mathcal {H}}}^k\lfloor M), \end{aligned}$$

We refer to [1, Theorem 2.15] for a proof.

#### Remark 2.2

- For $E=\{x_0\}$ and $E_{\varepsilon }=B_{\varepsilon }(x_0)$ ${\varepsilon }>0$ the weak convergence of measures (a) means for all $\varphi \in C^0(\overline{{\textsf{D}}})$:
  20 $$\begin{aligned} \int _{\textsf{D}}\mu _{E_{\varepsilon }} \varphi \;dx = \frac{1}{|B_{\varepsilon }(x_0)|}\int _{B_{\varepsilon }(x_0)} \varphi \;dx \rightarrow \varphi (x_0) \quad \text { as } {\varepsilon }\searrow 0. \end{aligned}$$
- For $E=\Gamma$ is a $(d-1)$-dimensional Lipschitz hypersurface with finite perimeter $\textrm{Per}(\Gamma )$, the weak convergence of measures (b) means for all $\varphi \in C^0(\overline{{\textsf{D}}})$:
  21 $$\begin{aligned} \int _{\textsf{D}}\mu _{E_{\varepsilon }} \varphi \;dx = \frac{1}{|E_{\varepsilon }|}\int _{E_{\varepsilon }} \varphi \;dx \rightarrow \frac{1}{\textrm{Per}(\Gamma )}\int _{\Gamma } \varphi \; d{{\mathcal {H}}}^{d-1} \quad \text { as } {\varepsilon }\searrow 0. \end{aligned}$$

#### Remark 2.3

When *E* is a hypersurface, we can actually prove that the convergence holds for the duality on $W^{1,p}({\textsf{D}})$. This means that (21) holds for every function $\varphi \in W^{1,p}({\textsf{D}})$, for $p\in [1,\infty )$, when we replace the last integral with $\int _\Gamma \textrm{Tr}_\Gamma (\varphi )\;d{{\mathcal {H}}}^{d-1}$, where $\textrm{Tr}_\Gamma$ is the trace operator on $\Gamma$.

Throughout the paper, we retain the notation $\mu _{E}$ for the limit measures given in Proposition 2.1.

#### Remark 2.4

(Lower dimensional inclusion) Of course, what we considered here was the removal of a *d*-dimensional object: $E_{\varepsilon }$ has nonempty interior. It is natural to wonder what might happen if we were to remove lower dimensional objects, for instance removing a centered disk in a three-dimensional object. We believe that our analysis would still be valid but, for the sake of readability, we stick with the removal of tubular neighborhoods.

***Notation***

In Definition 1.1 we have considered two types of perturbations, one consisting in adding some material outside of ${\Omega }$, the other in removing some material from ${\Omega }$. Naturally, this means that, depending on the case considered, either $\mu _{E_{\varepsilon }}$ or $-\mu _{E_{\varepsilon }}$ is involved in the linearised system. In order to alleviate notations and to not carry out moot distinctions, for every compact subset *E* such that $E\subset {\Omega }$ or $E\subset {\textsf{D}}\backslash {\overline{{\Omega }}}$ we define

22 $$\begin{aligned} \textrm{sgn}_{\Omega }(E):= {\left\{ \begin{array}{ll} +1 &{}\text { if }E\subset {\textsf{D}}\backslash {\overline{{\Omega }}}\,, \\ -1 &{}\text { if } E\subset {\Omega }. \end{array}\right. } \end{aligned}$$

### Topology Optimisation Problems for Semilinear Equations with Monotone Semilinearity

***Analytic Setting***

The first problem we tackle is that of a semilinear elliptic equation, where ${\Omega }\in {{\mathcal {A}}}({\textsf{D}})$ appears in the nonlinearity.

To be precise, we consider two coefficients $f_i\in {\textbf{R}}$ ($i=1,2$), as well as two nonlinearities $g_i=g_i(u)$ ($i=1,2$) that satisfy

23 $$\begin{aligned} g_i \text { is } C^1\text { increasing in }u,\text { and is globally bounded in } {\textbf{R}}_+~ (i=1,2). \end{aligned}$$

We then define, for every ${\Omega }\in {{\mathcal {A}}}({\textsf{D}})$, a nonlinearity $\rho _{\Omega }=\rho _{\Omega }(x,u)$ as

$$\begin{aligned} \rho _{\Omega }(x,u):=\chi _{\Omega }(x)g_1(u)+\chi _{{\textsf{D}}\backslash {\Omega }}(x)g_2(u). \end{aligned}$$

From [19, Theorem 4.4], if (23) is satisfied, then, for every ${\Omega }\in {{\mathcal {A}}}({\textsf{D}})$, the equation

24 $$\begin{aligned} \left\{ \begin{alignedat}{2} -\Delta u_{\Omega }+\rho _{\Omega }(x,u_{\Omega })&=f_1\chi _{\Omega }+f_2\chi _{{\textsf{D}}\backslash {\overline{{\Omega }}}}{} & {} \quad \text { in }{\textsf{D}}, \\ u_{\Omega }&=0{} & {} \quad \text { on }\partial {\textsf{D}}, \end{alignedat}\right. \end{aligned}$$

has a unique solution $u_{\Omega }\in H^1_0({\Omega })$. By standard elliptic regularity, for every $p\in [1,\infty )$, $u_{\Omega }\in W^{2,p}({\textsf{D}})$ so that $u_{\Omega }\in C^1({\overline{{\textsf{D}}}})$. We now study the topological state derivative of ${\Omega }\mapsto u_{\Omega }$. To give meaning to our afferent results, we need to lay down some basic definitions on the linearised system.

***Basic Computations***

Our subsequent analysis strongly hinges on the property of the linearised operator associated with (24). To justify the use of this linearisation, we simply observe that $U_{\varepsilon }:=\frac{u_{{\Omega }(E_{\varepsilon })}-u_{\Omega }}{|E_{\varepsilon }|}$ satisfies $U_{\varepsilon }=0$ on $\partial {\textsf{D}}$ and in a weak $W^{1,q}_0({\textsf{D}})$-sense, the following equation in ${\textsf{D}}$:

$$\begin{aligned}{} & {} -\Delta U_{{\varepsilon }}+\chi _{\Omega }\frac{(g_1(u_{{\varepsilon }})-g_1(u_{0})-g_2(u_{{\varepsilon }})+g_2(u_{0}))}{|E_{\varepsilon }|}+\frac{g_2(u_{{\varepsilon }})-g_2(u_0)}{|E_{\varepsilon }|}\\{} & {} \quad =\textrm{sgn}_{\Omega }(E)\left[ (g_2(u_{{\varepsilon }})-g_1(u_{{\varepsilon }}))+(f_1-f_2)\right] \mu _{E_{\varepsilon }}, \end{aligned}$$

where we used the simplified notation $u_{\varepsilon }:=u_{\Omega (E_{\varepsilon })}$ and $u_0:=u_{\Omega }$, and we should thus obtain, as ${\varepsilon }\searrow 0$, the following limit equation:

25 $$\begin{aligned} \begin{alignedat}{2} -\Delta U_0+\frac{\partial \rho _0}{\partial u}(x,u_0)&=\textrm{sgn}_{\Omega }(E)\left[ (g_2(u_0)-g_1(u_0))+(f_1-f_2)\right] \mu _E{} & {} \quad \text { in } {\textsf{D}}, \\ U_0&=0{} & {} \quad \text { on } \partial {\textsf{D}}, \end{alignedat} \end{aligned}$$

which has to be understood in a weak $W^{1,q}({\textsf{D}})$ sense for $q>d$ and will be explained in the next paragraph. In the following paragraph we give some background information about the linear operator used to define the linear equation on $U_0$.

***Notion of Weak Solution for the Linearised System***

The linearised operator associated with (24) is defined as

26 $$\begin{aligned} {\mathcal {L}}_{\Omega }:u\mapsto -\Delta u+\frac{\partial \rho _{\Omega }}{\partial u}(x,u_{\Omega })u. \end{aligned}$$

As we explained in Sect. 1.2, topological state derivatives “should”, in a sense made precise below, solve an equation of the form $-{\mathcal {L}}_{\Omega }u=\mu$ for some probability measure $\mu$, with homogeneous Dirichlet boundary conditions. Even in the case of the Laplacian there are natural Sobolev bounds on the regularity to be expected from solutions of such equations. This motivates the following definition.

#### Definition 2.5

Let ${\mathcal {M}}({\textsf{D}})$ be the set of Borel measures in *D* with finite total variation. Let $\mu \in {\mathcal {M}}({\textsf{D}})$. For every $q\in [1,\frac{d}{d-1})$, we say that a function $u\in W^{1,q}_0({\textsf{D}})$ is a weak $W^{1,q}_0$-solution of

27 $$\begin{aligned} \left\{ \begin{alignedat}{2} {\mathcal {L}}_{\Omega }u&=\mu{} & {} \quad \text { in }{\textsf{D}}, \\ u&=0{} & {} \quad \text { on }\partial {\textsf{D}}, \end{alignedat}\right. \end{aligned}$$

if, for every function $\varphi \in W^{1,q^\prime }_0({\textsf{D}})$ with $\frac{1}{q}+\frac{1}{q^\prime }=1$, there holds

28 $$\begin{aligned} \int _{\Omega }\nabla u\cdot \nabla \varphi \; dx+\int _{\Omega }\frac{\partial \rho _{\Omega }}{\partial u}(x,u_{\Omega }) u\varphi \;dx=\langle \varphi ,\mu \rangle , \end{aligned}$$

where the last duality bracket is to be understood in the sense of the duality between continuous functions and measures.

It should be noted that since $1\le q<\frac{d}{d-1}$, the conjugate Lebesgue exponent $q^\prime$ of *q*, $\frac{1}{q}+\frac{1}{q^\prime }=1$, satisfies $q^\prime >d$. From Sobolev embeddings this implies that the duality bracket $\langle \varphi ,\mu \rangle$ in (28) is well-defined. Definition 2.5 is a standard notion of weak solution for elliptic equations with measure data [22, 30].

As a first consequence of (23) we prove that (27) is well-posed.

#### Proposition 2.6

If $g_1, g_2$ satisfy (23) then, for every finite Borel measure $\mu$ in ${\textsf{D}}$, the equation (27) is well-posed: for every $q\in [1,\frac{d}{d-1})$ there exists a unique solution $u\in W^{1,q}_0({\textsf{D}})$ of (27). Furthermore, there exists a constant $C_q$ independent of $\mu$ such that

$$\begin{aligned} \Vert u\Vert _{W^{1,q}({\textsf{D}})}\le C_q \Vert \mu \Vert _{{\mathcal {M}}({\textsf{D}})}. \end{aligned}$$

It is obvious by the inclusion of the Lebesgue spaces $L^p$ that *u* does not depend on the exponent *q* and we abbreviate the first point of this proposition as “there exists a unique weak solution *u* to (27) that further satisfies that for every $q\in [1,\frac{d}{d-1})$, $u \in W^{1,q}_0({\textsf{D}})$.” Finally, observe that the Sobolev space in which (27) is well-posed depends on the space $\mu _E$ is in the dual of; in the case where $E=\{x_0\}$, $\mu _E$ is only in the dual space of $W^{1,q}({\textsf{D}})$, $q>d$. In the case $E=\Gamma$, $\mu _E$ is in the dual of all Sobolev spaces by the theory of Sobolev traces

***Expression of the Topological State Derivative***

Our main result here is the following theorem (recall that $\mu _{E}$ is defined in Proposition 2.1)

#### Theorem 2.7

Let $E\subset {\Omega }$ or $E\subset {\textsf{D}}\backslash \overline{{\Omega }}$ be either a point, $d-1$-dimensional Lipschitz surface or $1<k<d-1$ dimensional compact and smooth submanifold (without boundary). For every ${\varepsilon }>0$ we define $U_{\varepsilon }:=\frac{u_{{\Omega }(E_{\varepsilon })}-u_{\Omega }}{|E_{\varepsilon }|}$. Then, for every $q\in [1,\tfrac{d}{d-1})$,

$$\begin{aligned} U_{\varepsilon }\underset{{\varepsilon }\searrow 0}{\rightarrow }U_{0}\text { strongly in } W^{1,q}_0({\textsf{D}}), \end{aligned}$$

where $U_{0}$ is the unique solution to

29 $$\begin{aligned} \left\{ \begin{alignedat}{2} -\Delta U_{0}+\partial _u\rho _{\Omega }(x,u_{\Omega })U_{0}&=\textrm{sgn}_{\Omega }(E)\left\{ (g_2(u_{\Omega })-g_1(u_{\Omega }))+(f_1-f_2)\right\} \mu _{E}{} & {} \quad \text { in }{\textsf{D}}, \\ U_{0}&=0{} & {} \quad \text { on }\partial {\textsf{D}}. \end{alignedat}\right. \end{aligned}$$

Observe that, since $u_{\Omega }\in C^1({\overline{{\textsf{D}}}})$ and since $g_1, g_2$ are continuous, the product appearing on the right-hand side of (29) is indeed a Borel measure. In addition, $U_0$ depends on $\Omega$ and *E*

***Expression of Control Derivatives Using the Topological State Derivative***

We mentioned in the introduction of this paper that a control point of view allows to obtain the topological state derivative. Conversely, in low dimensions, the knowledge of the topological state derivatives enables the recovery of control derivatives. In this context, and to make our statement more precise, let us recall that, seeing $\chi _{\Omega }$ as a function in $L^2({\textsf{D}})$, we may extend the definition of $u_{\Omega }$ by defining, for every $f\in L^2({\textsf{D}})$, $u_f$ as the unique solution of (24) with $\chi _{\Omega }$ replaced with *f*, and $\chi _{{\textsf{D}}\backslash {\overline{{\Omega }}}}$ replaced with $(1-f)$. The $L^2$-differentiability of the map $f\mapsto u_f$ is standard. For every $h\in L^2({\textsf{D}})$, let

$$\begin{aligned} \dot{u}_{\chi _{\Omega },h}=\lim _{t\searrow 0}\frac{u_{\chi _{\Omega }+th}-u_{\chi _{\Omega }}}{t} \end{aligned}$$

be the directional derivative of $f\mapsto u_f$ at $\chi _{\Omega }$ in direction *h*. Recalling that ${\mathcal {L}}_{\Omega }$ was defined in (26), $\dot{u}_{\chi _{\Omega },h}$ solves (in the weak $H^{1}_0({\textsf{D}})$ sense):

30 $$\begin{aligned} \left\{ \begin{alignedat}{2} {\mathcal {L}}_{\Omega }\dot{u}_{\chi _{\Omega },h}&=h\left\{ (g_2(u_{\Omega })-g_1(u_{\Omega }))+(f_1-f_2)\right\}{} & {} \quad \text { in }{\textsf{D}}, \\ \dot{u}_{\chi _{\Omega },h}&=0{} & {} \quad \text { on } \partial {\textsf{D}}. \end{alignedat}\right. \end{aligned}$$

Our second theorem is the following:

#### Theorem 2.8

Assume $d\in \{2,3\}$. Then, for every $h\in L^2({\textsf{D}})$, $\dot{u}_{\chi _{\Omega },h}$ admits the following representation: for a.e. $x\in {\textsf{D}}$,

31 $$\begin{aligned} \dot{u}_{\chi _{\Omega },h}(x)=\int _{{\textsf{D}}} \textrm{sgn}_{\Omega }(y)U_{\{y\},0}(x)h(y)dy. \end{aligned}$$

where $U_{\{y\},0}$ is the solution of (27) with $\mu =\delta _y$.

Theorem 2.8 justifies the analogy between the topological state derivative and the Green kernel of the linearised operator. Of course, there is an interplay between the dimension assumption $d\in \{2,3\}$ and the integrability of the perturbation *h*. We also note here that we stated the theorem in the $L^2$ setting, as it is the most currently used for control derivatives.

### Asymptotic Expansion of $u_\Omega$ and Relation to the Topological State Derivative

***Asymptotic Analysis in the Linear Case***

In this section, we consider the model (24) with $g_1=g_2=0$ for point perturbations. To be precise we consider $u_{\varepsilon }\in H^1_0({\textsf{D}})$, such that

32 $$\begin{aligned} \int _{\textsf{D}}\nabla u_{\varepsilon }\cdot \nabla \varphi \;dx =\int _{\textsf{D}}(f_1 \chi _{\Omega _{\varepsilon }} + f_2\chi _{\Omega _{\varepsilon }^c})\varphi \;dx \quad \text { for all } \varphi \in H^1_0({\textsf{D}}). \end{aligned}$$

where

33 $$\begin{aligned} \Omega _{\varepsilon }&:= \Omega _{\varepsilon }(x_0,\omega ) := {\left\{ \begin{array}{ll} \Omega \cup \omega _{\varepsilon }(x_0) &{} \text { for }x_0\in {\textsf{D}}\setminus {\overline{\Omega }}, \\ \Omega \setminus \overline{\omega _{\varepsilon }(x_0)} &{} \text { for } x_0 \in \Omega , \end{array}\right. } \end{aligned}$$

and $\omega _{\varepsilon }(x_0):= x_0 + {\varepsilon }\omega$ with $\omega \subset {\textbf{R}}^d$ being a simply connected domain with $0\in \omega$. Furthermore, we define $u_{\varepsilon }:= u_{{\Omega }_{\varepsilon }(x_0,\omega )}$ for ${\varepsilon }>0$ and $u_0:= u_{\Omega }$.

The full asymptotic expansion for this equation including full topological expansions of several cost functionals has been studied in [26]. Recall the notation $T_{\varepsilon }(x) = x_0 + {\varepsilon }x$ for $x_0\in {\textsf{D}}{\setminus }\partial \Omega$. We now present a relation between the limit

34 $$\begin{aligned} U_0 := \lim _{{\varepsilon }\searrow 0} \frac{u_{\varepsilon }-u_0}{|\omega _{\varepsilon }|}, \end{aligned}$$

and the asymptotic expansion derived in [26] for $u_{\varepsilon }$, namely,

35 $$\begin{aligned} u_{\varepsilon }(x) = u_0(x) + {\varepsilon }^2( K(T_{\varepsilon }^{-1}(x)) + v(x) + \ln ({\varepsilon })b) +o({\varepsilon }^2) \quad \text { for } d=2, \end{aligned}$$

with $$b:= -\textrm{sgn}_{\Omega }(\{x_0\})(2\pi )^{-1}(f_1-f_2)$$ and

36 $$\begin{aligned} u_{\varepsilon }(x) = u_0(x) + {\varepsilon }^2( K(T_{\varepsilon }^{-1}(x)) + {\varepsilon }^{d-2}v(x)) + o({\varepsilon }^d) \quad \text { for } d\ge 3. \end{aligned}$$

Here *K* is a corrector function defined in ${\textbf{R}}^d$, while *v* is a boundary layer corrector defined in the bounded domain ${\textsf{D}}$. To be precise, *K* is given, in term of the fundamental solution $E(\cdot )$ of the Laplace operator $-\Delta$ in ${\textbf{R}}^d$, as

37 $$\begin{aligned} K(x) = \textrm{sgn}_{\Omega }(\{x_0\})(f_1-f_2)\int _\omega E(x-y)\; dy, \end{aligned}$$

with the fundamental solution being given by

38 $$\begin{aligned} E(x) = {\left\{ \begin{array}{ll} c_2 \ln (|x|) &{} \text { for } d=2, \\ c_d\frac{1}{|x|^{d-2}} &{} \text { for } d\ge 3, \end{array}\right. } \end{aligned}$$

with $c_2:=-\frac{1}{2\pi }$ and, if $d\ge 3$, $c_d:= ((d(d-2)\alpha (d))^{-1}$, $\alpha (d)$ denoting the volume of the unit ball in ${\textbf{R}}^d$. Thus, the function *K* satisfies

$$\begin{aligned} -\Delta K = \textrm{sgn}_{\Omega }(\{x_0\})(f_1-f_2)\chi _\omega \quad \text { in } {\textbf{R}}^d. \end{aligned}$$

and admits the following asymptotic expansion for $d\ge 2$:

39 $$\begin{aligned} K(x) = \textrm{sgn}_{\Omega }(\{x_0\})(f_1-f_2){\left\{ \begin{array}{ll} c_2 \ln (|x|) + O(|x|^{-1}) &{} \text { for } d=2, \\ c_d |x|^{-(d-2)} + O(|x|^{-(d-1)})&{} \text { for } d\ge 3, \end{array}\right. } \end{aligned}$$

so that when we denote by *R* the first term of the asymptotics of *K*, that is, $K(x) = R(x) + O(|x|^{d-1})$, we obtain

40 $$\begin{aligned} R(x) = \textrm{sgn}_{\Omega }(\{x_0\})(f_1-f_2){\left\{ \begin{array}{ll} c_2 \ln (|x|) &{} \text { for } d=2, \\ c_d |x|^{-(d-2)} &{} \text { for } d\ge 3. \end{array}\right. } \end{aligned}$$

The corrector function $v\in H^1({\textsf{D}})$ satisfies

41 $$\begin{aligned} \left\{ \begin{alignedat}{2} -\Delta v&=0{} & {} \quad \text { in } {\textsf{D}},\\ v(x)&= -R(x-x_0){} & {} \quad \text { on } \partial {\textsf{D}}. \end{alignedat}\right. \end{aligned}$$

To state the main result, let us briefly recall the setting of [26]. Since the result in [26] was provided only for $d\in \{2,3\}$, we give a short proof in the appendix.

#### Lemma 2.9

Introduce the function

42 $$\begin{aligned} K_{\varepsilon }= \frac{(u_{\varepsilon }-u_0)\circ T_{\varepsilon }}{{\varepsilon }^2}, \quad {\varepsilon }>0. \end{aligned}$$

Set ${\textsf{D}}_{\varepsilon }:= T_{\varepsilon }^{-1}({\textsf{D}})$ for ${\varepsilon }>0$. Then there is a constant $C=C_{p,d}>0$, which depends on *p* and *d*, such that for $d=2$:

43 $$\begin{aligned} \Vert {\varepsilon }(K_{\varepsilon }- K - v\circ T_{\varepsilon }-\ln ({\varepsilon })b)\Vert _{L^2({\textsf{D}}_{\varepsilon })} + \Vert \nabla (K_{\varepsilon }- K - v\circ T_{\varepsilon }-\ln ({\varepsilon })b)\Vert _{L^2({\textsf{D}}_{\varepsilon })^d} \le C {\varepsilon }, \end{aligned}$$

with $b:=-\textrm{sgn}_{\Omega }(\{x_0\}) \frac{1}{2\pi }(f_1-f_2)$ and for $d\ge 3$:

44 $$\begin{aligned} \Vert {\varepsilon }(K_{\varepsilon }- K - {\varepsilon }^{d-2}v\circ T_{\varepsilon })\Vert _{L^2({\textsf{D}}_{\varepsilon })} + \Vert \nabla (K_{\varepsilon }- K - {\varepsilon }^{d-2}v\circ T_{\varepsilon })\Vert _{L^2({\textsf{D}}_{\varepsilon })^d} \le C {\varepsilon }^{\frac{d}{2}}. \end{aligned}$$

***Relation Between Asymptotic Expansion and Topological State Derivative*** To draw a connection to the topological state derivative, let us note that we can write the expansions (43) and (44) pointwise as (35) and (36). Our main result is that the estimate (44) in fact implies the following estimate:

#### Theorem 2.10

- For $x_0\in {\textsf{D}}\setminus \partial \Omega$ and $\omega \subset {\textbf{R}}^d$ be a simply connected and bounded domain with $0\in \omega$ we use the definition of $\Omega _{\varepsilon }(x_0,\omega )$ of (11) and set $u_{\varepsilon }:= u_{\Omega _{\varepsilon }(x_0,\omega )}$, $u_0:= u_\Omega$ and
  45 $$\begin{aligned} U_{\varepsilon }:= \frac{u_{\varepsilon }- u_0}{|\omega _{\varepsilon }|}, \quad {\varepsilon }>0. \end{aligned}$$
  Let *K* and *v* be defined by (37) and (41), respectively. Then we have the following results.
- (i) From the asymptotic expansion (43) and (44) we conclude that the limit (34) exists. In fact we have
  46 $$\begin{aligned} U_0(x) = \textrm{sgn}_{\Omega }(\{x_0\})(f_1-f_2) E(x-x_0) + v(x), \quad \text { for a.e. } x\in {\textsf{D}}, \end{aligned}$$
  and for a.e. $x\in {\textsf{D}}$, we have with $b:= -\textrm{sgn}_{\Omega }(\{x_0\})(2\pi )^{-1}(f_1-f_2)$:
  47 $$\begin{aligned} U_0(x) = \lim _{{\varepsilon }\searrow 0}\frac{1}{|\omega _{\varepsilon }|} {\varepsilon }^2(K(T_{\varepsilon }^{-1}(x)) + v(x) + \ln ({\varepsilon })b) \quad \text { for } d=2, \end{aligned}$$
  and
  48 $$\begin{aligned} U_0(x) = \lim _{{\varepsilon }\searrow 0}\frac{1}{|\omega _{\varepsilon }|} {\varepsilon }^2 (K(T_{\varepsilon }^{-1}(x)) + {\varepsilon }^{d-2} v(x)) \quad \text { for } d\ge 3. \end{aligned}$$
- (ii) Let the space dimension be $d=2$. Then there exist constant $C=C_{p,d}>0$, which depends on *p* and *d*, such that
  - $\bullet$ We have for all $p\in (2,\infty )$:
    49 $$\begin{aligned} \Vert U_{\varepsilon }- U_0\Vert _{L^p({\textsf{D}})} \le C{\varepsilon }^{\frac{2}{p}}. \end{aligned}$$
  - $\bullet$ We have for all $p\in (1,2)$:
    50 $$\begin{aligned} \Vert U_{\varepsilon }- U_0\Vert _{W^{1,p}({\textsf{D}})} \le C{\varepsilon }^{\frac{2}{p}-1}. \end{aligned}$$
- (iii) Let the space dimension be $d\ge 3$. Then there exists $C=C_{p,d}>0$, which also depends on *p* and *d*, such that:
  - $\bullet$ We have for all $p\in \left( \frac{d}{d-1},\frac{d}{d-2}\right)$:
    51 $$\begin{aligned} \Vert U_{\varepsilon }- U_0\Vert _{L^p({\textsf{D}})} \le C{\varepsilon }^{\frac{d-p(d-2)}{p}}. \end{aligned}$$
  - $\bullet$ We have for all $p\in (1,\frac{d}{d-1})$,
    52 $$\begin{aligned} \Vert U_{\varepsilon }- U_0\Vert _{W^{1,p}({\textsf{D}})} \le C{\varepsilon }^{\frac{d-p(d-1)}{p}}. \end{aligned}$$
    Notice that $$p^{-1}(d-p(d-1)) \in (0,1)$$.

The following corollary shows that, if $\omega =B_1(0)$ is the unit ball centered at the origin, then the convergence rate of $U_{\varepsilon }$ to $U_0$ in the $L^p({\textsf{D}})$ norm can be improved to an order between (1, 2).

#### Corollary 2.11

Assume that $\omega =B_1(0)$ is the unit ball in ${\textbf{R}}^d$ centered at the origin. Then we have for all $p\in (1,\frac{d}{d-2})$ for $d\ge 3$ or $p\in (1,\infty )$ for $d=2$,

53 $$\begin{aligned} \Vert U_{\varepsilon }- U_0\Vert _{L^p({\textsf{D}})} \le C{\varepsilon }^{\frac{d-p(d-2)}{p}}. \end{aligned}$$

That means the convergence rates (50) for $d=2$ and (52) for $d\ge 3$ are improved for all $p\in (1,\frac{d}{d-1})$.

Let us finish this section with two remarks.

#### Remark 2.12

The function $U_0(x):= |\omega |^{-1}(R(x-x_0) + v(x))\in W^{1,p}_0({\textsf{D}})$ with $p\in [1,\frac{d}{d-1})$ solves in a weak sense:

54 $$\begin{aligned} \left\{ \begin{alignedat}{2} -\Delta U_0&= \textrm{sgn}_{\Omega }(\{x_0\})(f_1-f_2) \delta _{x_0}{} & {} \quad \text { in } {\textsf{D}}, \\ U_0&=0{} & {} \quad \text { on } \partial {\textsf{D}}. \end{alignedat}\right. \end{aligned}$$

The decomposition of the solution $U_0$ of (54) in a regular part $|\omega |^{-1}v$ and a singular part $|\omega |^{-1}R(x-x_0) = \textrm{sgn}_{\Omega }(\{x_0\})(f_1-f_2)|\omega | E(x-x_0)$ is well-known and often used in the numerical investigation of this type of equation [28, 29].

#### Remark 2.13

The improved $L^p({\textsf{D}})$ convergence rate of Corollary 2.11 is a result of the symmetry of the inclusion $\omega =B_1(0)$. We note that (53) implies for $p>1$ close to one that

55 $$\begin{aligned} \Vert (U_{\varepsilon }- U_0)/{\varepsilon }\Vert _{L^p({\textsf{D}})} = o(1) \end{aligned}$$

and thus $U_{\varepsilon }^2:= (U_{\varepsilon }- U_0)/{\varepsilon }\rightarrow 0$ strongly in $L^p({\textsf{D}})$ for *p* close to one. This is actually consistent with the limit equation of $U_{\varepsilon }^2$. To see this we recall that

$$\begin{aligned} U_{\varepsilon }=\frac{u_{\varepsilon }-u_0}{|\omega _{\varepsilon }|}, \quad U_{\varepsilon }^2:= \frac{U_{\varepsilon }-U_0}{{\varepsilon }}, \quad {\varepsilon }>0. \end{aligned}$$

It is readily checked that $U_{\varepsilon }^2$ satisfies for all $\varphi \in H^1_0({\textsf{D}})$:

56 $$\begin{aligned} \int _{\textsf{D}}\nabla U_{\varepsilon }^2\cdot \nabla \varphi \;dx = \text {sgn}_\Omega (\{x_0\}) (f_1-f_2)\frac{1}{|\omega _{\varepsilon }|} \int _{\omega _{\varepsilon }} {\varepsilon }^{-1}(\varphi - \varphi (x_0)) \;dx. \end{aligned}$$

Now changing variables on the right hand side and integrating by parts on the left hand side, we obtain for $\varphi \in C^2_c({\textsf{D}})$:

57 $$\begin{aligned} -\int _{\textsf{D}}U_{\varepsilon }^2 \Delta \varphi \;dx = \text {sgn}_\Omega (\{x_0\}) (f_1-f_2) \int _{\omega } {\varepsilon }^{-1}(\varphi (x_0+{\varepsilon }x) - \varphi (x_0)) \;dx. \end{aligned}$$

Hence, if $U_{\varepsilon }^2\rightarrow U_0^2$ in $L_p({\textsf{D}}$), then passing to the limit yields for $\varphi \in C^2_c({\textsf{D}})$:

58 $$\begin{aligned} -\int _{\textsf{D}}U^2_0 \Delta \varphi \;dx = \text {sgn}_\Omega (\{x_0\}) (f_1-f_2) \int _\omega \nabla \varphi (x_0)\cdot x\;dx. \end{aligned}$$

So we observe that for $\omega =B_1(0)$ the integral on the right hand side vanishes due to the symmetry of $B_1(0)$. This is consistent with $U_0^2=0$ so that also the left hand side is zero.

***Topological State Derivative Via the Formal Asymptotic Expansion of the Semilinear Equation***

We consider the semilinear equation (24) with right hand side $f\in L^2({\textsf{D}})$:

59 $$\begin{aligned} \left\{ \begin{alignedat}{2} -\Delta u_{\Omega }+\rho _{\Omega }(x,u_{\Omega })&=f{} & {} \quad \text { in }{\textsf{D}}, \\ u_{\Omega }&=0{} & {} \quad \text { on }\partial {\textsf{D}}. \end{alignedat}\right. \end{aligned}$$

From Theorem 2.7 we have that the limit

60 $$\begin{aligned} U_0 := \lim _{{\varepsilon }\searrow 0 }\frac{u_{\varepsilon }- u_0}{|\omega _{\varepsilon }|}, \end{aligned}$$

satisfies61We now want to show that this limit can also be obtained using an asymptotic analysis of $u_{\Omega }$. For this purpose, we can split the solution $U_0$ into an irregular part $|\omega |^{-1}R$ and regular part $|\omega |^{-1}v$ as follows (the factor $|\omega |^{-1}$ is chosen to make the link between the asymptotic expansion and will become clear shortly). Set $g_{x_0}:= g_2(u_{\Omega }(x_0))-g_1(u_{\Omega }(x_0))$ and let *R* be defined by

62 $$\begin{aligned} R(x) = \textrm{sgn}_{\Omega }(\{x_0\}) g_{x_0}|\omega |{\left\{ \begin{array}{ll} c_2 \ln (|x|) &{} \text { for } d=2, \\ c_3 |x|^{-1} &{} \text { for } d= 3. \end{array}\right. } \end{aligned}$$

Then we have in a distributional sense:

63 $$\begin{aligned} -\Delta (R(x-x_0)) = |\omega | \textrm{sgn}_{\Omega }(\{x_0\}) g_{x_0} \delta _{x_0} \quad \text { in } {\textbf{R}}^d. \end{aligned}$$

Now we define $v\in H^1({\textsf{D}})$ as follows64Notice that in comparison to the linear setting studied in the previous section we now have an additional term on the right hand side, namely, $R(x-x_0)\partial _u\rho _{\Omega }(x,u_{\Omega })$, which accounts for the fact that the equation (63) does not have a lower order term. Then it is readily checked that

65 $$\begin{aligned} U_0(x) := |\omega |^{-1}(R(x-x_0) + v(x)), \quad \text { a.e. } x\in {\textsf{D}}, \end{aligned}$$

solves the equation (61).

We now show that $U_0$ can be obtained from the asymptotics of $u_{\Omega }$. We let $\omega$, $x_0$ and $\Omega _{\varepsilon }= \Omega _{\varepsilon }(x_0,\omega )$ be as in the previous section. Denote again the solution of (59) $u_{{\Omega }_{\varepsilon }}$ by $u_{\varepsilon }$ and $u_0:= u_{\Omega }$. Following the formal asymptotic expansion of [31] we have the following expansion of $u_{{\Omega }_{\varepsilon }}$:

66 $$\begin{aligned} u_{\varepsilon }(x) = u_0(x) + {\varepsilon }^2( K(T_{\varepsilon }^{-1}(x)) + v(x) + \ln ({\varepsilon })b) +o({\varepsilon }^2) \quad \text { for } d=2, \end{aligned}$$

with $b:= - \textrm{sgn}_{\Omega }(\{x_0\}) g_{x_0} |\omega |(2\pi )^{-1}$ and for $d=3$

67 $$\begin{aligned} u_{\varepsilon }(x) = u_0(x) + {\varepsilon }^2( K(T_{\varepsilon }^{-1}(x)) + {\varepsilon }v(x)) + o({\varepsilon }^3) \quad \text { for } d=3. \end{aligned}$$

Here *K*, given by

68 $$\begin{aligned} K(x) = \textrm{sgn}_{\Omega }(\{x_0\}) g_{x_0} \int _\omega E(x-y)\;dy, \end{aligned}$$

solves for $d\in \{2,3\}$ the equation

69 $$\begin{aligned} -\Delta K = \textrm{sgn}_{\Omega }(\{x_0\}) g_{x_0} \chi _{\omega } \quad \text { in } {\textbf{R}}^d. \end{aligned}$$

#### Theorem 2.14

For $x_0\in {\textsf{D}}\setminus \partial \Omega$ and $\omega \subset {\textbf{R}}^d$ with $0\in \omega$ we use the definition of $\Omega _{\varepsilon }(x_0,\omega )$ of (11) and set $u_{\varepsilon }:= u_{\Omega _{\varepsilon }(x_0,\omega )}$ and $u_0:= u_\Omega$ and

70 $$\begin{aligned} U_{\varepsilon }:= \frac{u_{\varepsilon }- u_0}{|\omega _{\varepsilon }|}, \quad {\varepsilon }>0. \end{aligned}$$

Let *K* and *v* be defined by (68) and (64), respectively. Then we have the following results. From the asymptotic expansion (66) and (67), we conclude that the limit (60) exists. In fact we have

71 $$\begin{aligned} U_0(x) = |\omega |^{-1}(R(x-x_0) + v(x)), \quad \text { for a.e. } x\in {\textsf{D}}, \end{aligned}$$

and for a.e. $x\in {\textsf{D}}$, we have with $b:= - \textrm{sgn}_{\Omega }(\{x_0\})|\omega |g_{x_0}(2\pi )^{-1}$:

72 $$\begin{aligned} U_0(x) = \lim _{{\varepsilon }\searrow 0}\frac{1}{|\omega _{\varepsilon }|} {\varepsilon }^2(K(T_{\varepsilon }^{-1}(x)) + v(x) + \ln ({\varepsilon })b) \quad \text { for } d=2, \end{aligned}$$

and

73 $$\begin{aligned} U_0(x) = \lim _{{\varepsilon }\searrow 0}\frac{1}{|\omega _{\varepsilon }|} {\varepsilon }^2 (K(T_{\varepsilon }^{-1}(x)) + {\varepsilon }v(x)) \quad \text { for } d= 3. \end{aligned}$$

The proof of this theorem follows the lines of the proof of item (i) of Theorem 2.10.

## Main Results for Operator Point Perturbation in Linear Transmission Problems

In this section we show how our type of analysis carries on to transmission problems, which is also referred to as “perturbation of the operator”. The analysis of this type of perturbation is more difficult than perturbations of lower order terms and typically involves so-called “polarisation matrices”; [6, 12, 32]. We will see that the topological state derivative for point perturbations of the operator exists, but exhibits a very low regularity.

### Topological State Derivative for the Transmission Problem

***Analytic Set-Up***

Throughout this section we fix a set ${\Omega }\in {{\mathcal {A}}}({\textsf{D}})$ and assume $\Omega \Subset {\textsf{D}}$ is smooth. For two fixed parameters $\beta _1, \beta _2>0$ and every ${\Omega }\in {{\mathcal {A}}}({\textsf{D}})$, we define

$$\begin{aligned} \beta _{\Omega }:=\beta _1\chi _{\Omega }+\beta _2\chi _{{\textsf{D}}\backslash {\overline{{\Omega }}}}. \end{aligned}$$

Now for $f\in L^p({\textsf{D}})$ with $p>d$, let $u_{\Omega }$ be the unique weak solution in $W^{1,p}({\textsf{D}})$ of the equation74We note that the strong form of this equation reads: denoting by $u^+:=u_{|\Omega }$ and $u^-:= u_{|{\textsf{D}}{\setminus }{\overline{\Omega }}}$, we have75***Topological Perturbation Under Consideration***

In this section, we consider only point perturbations $\Omega _{\varepsilon }(x_0,\omega )$ at points $x_0\in {\textsf{D}}{\setminus }\partial \Omega$ of the set $\Omega$ defined by

76 $$\begin{aligned} \Omega _{\varepsilon }&:= \Omega _{\varepsilon }(x_0,\omega ) := {\left\{ \begin{array}{ll} \Omega \cup \omega _{\varepsilon }(x_0) &{} \text { for }x_0\in {\textsf{D}}\setminus {\overline{\Omega }}, \\ \Omega \setminus \overline{\omega _{\varepsilon }(x_0)} &{} \text { for } x_0 \in \Omega , \end{array}\right. } \end{aligned}$$

and $\omega _{\varepsilon }(x_0):= x_0 + {\varepsilon }\omega$ with $\omega \subset {\textbf{R}}^d$ being a simply connected domain with $0\in \omega$. We note that the special case $\omega =B_1(0)$ would correspond to the dilatation $\Omega (E_{\varepsilon })$ for $E=\{x_0\}$ considered in the previous sections. Following [9, 12] we decided to treat more general perturbations.

As we will see, the right hand side of the limit equation $U_0$ for the transmission problem will involve the divergence of a measure. As this is the only part which can not be covered by the techniques thus far used in studying semilinear models, we devote a paragraph to some basic definitions.

***Very Weak Solutions for Elliptic Equations with Divergence-of-Measure Right Hand Side***

We consider, for a given $\zeta \in {\mathbb {R}}^d$, the Dirac measure $\mu := \zeta \delta _{x_0} \in {\mathcal {M}}({\textsf{D}})^d$ concentrated at $x_0\in {\textsf{D}}\setminus \partial \Omega$, the equation77The weak formulation would read

78 $$\begin{aligned} \int _{\textsf{D}}\beta _{\Omega }\nabla \varphi _\mu \cdot \nabla \varphi \;dx = \zeta \cdot \nabla \varphi (x_0)\quad \text { for all } \varphi \in H^1_0({\textsf{D}}), \end{aligned}$$

which is obviously not well-defined. At this point, let us observe that by interior regularity $\nabla u_{\Omega }$ is continuous in a neighborhood of the perturbation point $x_0\in {\textsf{D}}{\setminus }\partial \Omega$. Furthermore, we also need to ensure that $\nabla \varphi$ is continuous as well. We thus resort to a notion of weak solution reminiscent of the one introduced in [23]: for every $p>d$, for $v\in L^p({\textsf{D}})$, let $\varphi _v \in W^{2,p}({\Omega }\cup ({\textsf{D}}{\setminus }\overline{{\Omega }}))\cap H^1_0({\textsf{D}})$ be the unique solution of79The well-posedness of this equation follows from arguments similar to the ones used in the proof of Proposition 5.1. By elliptic regularity, for every $p>d$, we have, for two constants $C_p, C_p'$

80 $$\begin{aligned} \Vert \varphi _v\Vert _{C^1({\Omega }\cup ({\textsf{D}}\setminus {\Omega }))} \le C_p\Vert \varphi _v\Vert _{W^{2,p}({\Omega }\cup ({\textsf{D}}\setminus {\Omega }))} \le C_p'\Vert v\Vert _{L^p({\textsf{D}})} \end{aligned}$$

for all $v\in L^p({\textsf{D}})$. Now choosing $\varphi _v$ in (78) and integrating by parts we obtain

81 $$\begin{aligned} \int _{\textsf{D}}u_\Omega v\; dx = \int _{\textsf{D}}\beta _\Omega \nabla u_\Omega \cdot \nabla \varphi _v\;dx =\int _{\textsf{D}}\langle \nabla u_{\Omega },\nabla \varphi _v\rangle d\mu = \zeta \cdot \nabla \varphi _v(x_0) \end{aligned}$$

for all $v\in L^p({\textsf{D}})$. We note that the interface terms vanish in view of the choice of the test function $\varphi _v$. This leads to the following definition:

#### Definition 3.1

Let $q\in [1,\frac{d}{d-1})$ and $q^\prime$ be its conjugate Lebesgue exponent. We say that $\phi _\mu$ is a very weak $W^{1,q}_0({\textsf{D}})$-solution of (78) if

82 $$\begin{aligned} \int _{\textsf{D}}\phi _\mu v = \nabla \varphi _v(x_0)\cdot \zeta \quad \text { for all } v\in L^{q^\prime }({\textsf{D}}), \end{aligned}$$

where $\varphi _v\in W^{1,q^\prime }_0({\textsf{D}})$ solves83It is important to note that the $\Omega$ dependence is now transferred to the definition of $\varphi _v$.

The main proposition is the following:

#### Proposition 3.2

Let $x_0\in {\textsf{D}}{\setminus }\partial \Omega$. For $\zeta \in {\textbf{R}}^d$ let $\mu := \zeta \delta _{x_0}$. The equation84has a unique very weak solution in the sense of Definition 3.1. Furthermore, for every $q\in [1,\frac{d}{d-1})$, there exists a constant $C_q>0$ such that

$$\begin{aligned} \Vert \phi _{\mu }\Vert _{L^q({\textsf{D}})}\le C_q\Vert \mu \Vert _{{\mathcal {M}}({\textsf{D}})}. \end{aligned}$$

***Expression of the Topological State Derivative***

Our main theorem is the following:

#### Theorem 3.3

Let $\Omega \in {{\mathcal {A}}}({\textsf{D}})$ with $\Omega \Subset {\textsf{D}}$. Let $x_0,\omega$ and $\Omega _{\varepsilon }(x_0,\omega )$ be as in (76) and denote by $u_{\Omega }$ the unique weak solution to (74). We have that $U_{\varepsilon }=\frac{u_{\Omega _{\varepsilon }(x_0,\omega )}-u_\Omega }{|\omega _{\varepsilon }|}$ is bounded in $L^q({\textsf{D}})$ for $q\in [1,\frac{d}{d-1})$ and we have for a constant $C=C_{q,d}>0$, depending on *q* and *d*:

85 $$\begin{aligned} \Vert U_{\varepsilon }- U_0\Vert _{L^q({\textsf{D}})} \le C({\varepsilon }^{\frac{d-q(d-1)}{q}} + \Vert K_{\varepsilon }- K\Vert _{L^1(\omega )}) \end{aligned}$$

and thus in particular $U_{\varepsilon }\rightarrow U_0$ strongly in $L^q({\textsf{D}})$ as ${\varepsilon }\searrow 0$. The limit $U_0$ solves:

86 $$\begin{aligned} \int _{\textsf{D}}U_0 v \;dx = \textrm{sgn}_{\Omega }(\{x_0\}) (\beta _2-\beta _1)\left( \frac{1}{|\omega |}\int _\omega \nabla K + \nabla u_0(x_0)\; dx\right) \cdot \nabla \varphi _v(x_0) \end{aligned}$$

for all $v\in L^{q^\prime }({\textsf{D}})$, where $\varphi _v$ is the solution to (79). Here, *K* belongs to the Beppo-Levi space $\dot{\text {BL}}({\textbf{R}}^d)$^1^ and is the unique solution to:

87 $$\begin{aligned} \int _{{\textbf{R}}^d} \beta _\omega \nabla K\cdot \nabla \varphi \;dx = \textrm{sgn}_{\Omega }(\{x_0\})(\beta _2-\beta _1)\int _\omega \nabla u_0(x_0)\cdot \nabla \varphi \;dx, \end{aligned}$$

for all $$\varphi \in \dot{\text {BL}}({\textbf{R}}^d)$$.

#### Remark 3.4

(Polarisation matrix) We note that $K=K[\nabla u_0(x_0)]$ actually depends linearly on the vector $\nabla u_0(x_0)$ through the equation (87). Consequently, the map

88 $$\begin{aligned} \nabla u_0(x_0) \mapsto \frac{1}{|\omega |} \int _\omega \nabla K \;dx: {\textbf{R}}^d\rightarrow {\textbf{R}}^d, \end{aligned}$$

is also linear and thus there is a so-called polarisation matrix $A_\omega \in {\textbf{R}}^d$; see [12, 32], which depends on $\beta _1,\beta _2$ and $\omega$, such that

89 $$\begin{aligned} A_\omega \nabla u_0(x_0) = \frac{1}{|\omega |} \int _\omega \nabla K \;dx. \end{aligned}$$

It follows that the equation (86) is equivalent to

90 $$\begin{aligned} \int _{\textsf{D}}U_0 v \;dx = \textrm{sgn}_{\Omega }(\{x_0\}) (\beta _2-\beta _1) (A_\omega +I_d)\nabla u_0(x_0)\cdot \nabla \varphi _v(x_0) \quad \text { for all } v\in L^p({\textsf{D}}), \end{aligned}$$

where $I_d\in {\textbf{R}}^{d\times d}$ denotes the identity matrix.Considering the special case $\omega =B_1(0)$, one readily checks that the solution *K* of equation (87) is given by

91 $$\begin{aligned} K(x)={\left\{ \begin{array}{ll}C_\beta \nabla u_0(x_0)\cdot x,\quad &{}\text { for } x\in \omega ,\\ C_\beta \nabla u_0(x_0)\cdot \frac{x}{|x|^d},\quad &{}\text { for }x\in {\textbf{R}}^d\setminus {\overline{\omega }},\end{array}\right. } \end{aligned}$$

where $C_\beta :=\textrm{sgn}_{\Omega }(\{x_0\})(\beta _2-\beta _1)(\beta _1-\beta _2-d\beta _2)^{-1}$. Thus, for the unit ball inclusion, the polarisation matrix is given by $A_\omega =C_\beta I_d$.

### Asymptotic Expansion of $u_\Omega$ and the Relation to the Topological State Derivative

We start this section by giving some results regarding the asymptotic expansion of $u_{\Omega _{\varepsilon }(x_0,\omega )}$. Note that these are derived using compound asymptotics; see [6, 11, 24].

***Asymptotic Analysis of***  $u_\Omega$

Let $x_0\in {\textsf{D}}{\setminus }\partial \Omega$, $\omega \subset {\textbf{R}}^d$ a simply connected and bounded domain with $0\in {\textbf{R}}^d$ and set $u_{\varepsilon }:= u_{\Omega _{\varepsilon }(x_0,\omega )}$, where $\Omega _{\varepsilon }(x_0,\omega )$ is as defined in (11). For ${\varepsilon }>0$ we introduce

92 $$\begin{aligned} K_{\varepsilon }:= \frac{(u_{\varepsilon }- u_0)\circ T_{\varepsilon }}{{\varepsilon }}. \end{aligned}$$

It is a classical result that the limit of $K_{\varepsilon }$ is in fact the unique solution to: find $K\in \dot{\text {BL}}({\textbf{R}}^d)$ such that

93 $$\begin{aligned} \int _{{\textbf{R}}^d} \beta _\omega \nabla K\cdot \nabla \varphi \;dx =\textrm{sgn}_{\Omega }(\{x_0\})(\beta _2-\beta _1)\int _\omega \nabla u_0(x_0)\cdot \nabla \varphi \;dx, \end{aligned}$$

for all $\varphi \in \dot{\text {BL}}({\textbf{R}}^d)$. The function *K* admits the asymptotics

94 $$\begin{aligned} K(x) = R(x) + O\left( \frac{1}{|x|^d}\right) . \end{aligned}$$

To state the final asymptotic expansion we need to introduce the regular boundary corrector *v* compensating the error introduced by *K* on $\partial {\textsf{D}}$.

The corrector $v\in H^1({\textsf{D}})$ is defined as the unique solution to $v(x)=-R(x-x_0)$ on $\partial {\textsf{D}}$ and

95 $$\begin{aligned} \int _{\textsf{D}}\beta _\Omega \nabla v \cdot \nabla \varphi \;dx = \textrm{sgn}_{\Omega }(\{x_0\})(\beta _2-\beta _1)\int _{\partial \Omega } \partial _\nu R(x-x_0) \varphi (x) \;dx \end{aligned}$$

for all $$\varphi \in H^1_0({\textsf{D}})$$.

The following lemma states the main result regarding the first order asymptotic expansion. We closely follow the arguments of [11, Theorem 3.15], but since we require estimates in $L^q$, we provide the main steps of the proof in the appendix.

#### Lemma 3.5

For $q\in (1,\frac{d}{d-1})$ there is a constant $C=C_{q,d}>0$, which depends on *q* and *d*, such that for all ${\varepsilon }>0$ small:

96 $$\begin{aligned} \Vert {\varepsilon }(K_{\varepsilon }- K - {\varepsilon }^{d-1} v\circ T_{\varepsilon })\Vert _{L^q({\textsf{D}}_{\varepsilon })} + \Vert \nabla (K_{\varepsilon }- K - {\varepsilon }^{d-1}v\circ T_{\varepsilon })\Vert _{L^q({\textsf{D}}_{\varepsilon })^d} \le C{\varepsilon }, \end{aligned}$$

which, by considering the scaling of the norms and ${\textsf{D}}_{\varepsilon }= T^{-1}_{\varepsilon }({\textsf{D}})$, is equivalent to

97 $$\begin{aligned} \Vert K_{\varepsilon }\circ T_{\varepsilon }^{-1} - K\circ T_{\varepsilon }^{-1} - {\varepsilon }^{d-1} v\Vert _{W^{1,q}({\textsf{D}})} \le C{\varepsilon }^{\frac{d}{q}}. \end{aligned}$$

***Relation Between Asymptotic Expansion and Topological State Derivative***

We now want to make the connection between the topological expansion and the topological state derivative. For this we note that the estimate (96) reads on the fixed domain ${\textsf{D}}$:

98 $$\begin{aligned} u_{\varepsilon }(x) = u_0(x) + {\varepsilon }K(T_{\varepsilon }^{-1}(x)) + {\varepsilon }^d v(x) + o({\varepsilon }^d;x), \end{aligned}$$

where $o({\varepsilon }^d;x)$ denotes $o({\varepsilon }^d)$ almost everywhere in ${\textsf{D}}$. To see the relation between the topological state derivative $U_0$ of Theorem 3.3 and the asymptotic expansion (Lemma (3.5)), we first note that the equation (93) can be written as follows

99 $$\begin{aligned} \begin{aligned} \int _{{\textbf{R}}^d} \nabla K\cdot \nabla \varphi \;dx&=\textrm{sgn}_{\Omega }(\{x_0\})\frac{\beta _2-\beta _1}{\beta (x_0)}\bigg (\int _\omega \nabla u_0(x_0)\cdot \nabla \varphi \;dx \\&\quad + \int _\omega \nabla K \cdot \nabla \varphi \;dx \bigg ), \end{aligned} \end{aligned}$$

where $\beta (\cdot )$ is a piecewise constant function defined by

$$\begin{aligned} \beta (x_0)={\left\{ \begin{array}{ll} \beta _2 &{} \text { for } x_0\in {\textsf{D}}\setminus {\overline{\Omega }}, \\ \beta _1 &{} \text { for } x_0 \in \Omega . \end{array}\right. } \end{aligned}$$

Therefore, with $E(\cdot )$ denoting the fundamental solution of $-\Delta$ on ${\textbf{R}}^d$, it is a classical result that *K* can be expressed as follows

$$\begin{aligned} K(x)&= \textrm{sgn}_{\Omega }(\{x_0\})\frac{\beta _2-\beta _1}{\beta (x_0)}\bigg (\int _\omega \nabla u_0(x_0)\cdot \nabla E(x-y) \;dy \\&\quad + \int _\omega \nabla K(y) \cdot \nabla E(x-y) \;dy \bigg ). \end{aligned}$$

Therefore, performing a Taylor expansion, we see that the first asymptotic term of *K*(*x*) as $|x|\rightarrow \infty$ can be written as

100 $$\begin{aligned} R(x) = \underbrace{\textrm{sgn}_{\Omega }(\{x_0\})\frac{\beta _2-\beta _1}{\beta (x_0)}\left( \int _\omega \nabla u_0(x_0) \;dy + \int _\omega \nabla K(y) \;dy \right) }_{\xi _{x_0}}\cdot \nabla E(x), \end{aligned}$$

where $\xi _{x_0}$ is a vector depending on $\nabla u_0(x_0)$. Notice that $\xi _{x_0}$ can also be expressed through the polarisation matrix $A_\omega$ of Remark 3.4 as follows

101 $$\begin{aligned} \xi _{x_0} = \textrm{sgn}_{\Omega }(\{x_0\})\frac{\beta _2-\beta _1}{\beta (x_0)}|\omega | (A_\omega +I_d)\nabla u_0(x_0)\cdot \nabla E(x) \end{aligned}$$

making the dependence on $x_0$ more explicit. Now we note that the function $U_0(x):= |\omega |^{-1}(\xi _{x_0}\cdot \nabla E(x-x_0) + v(x))$ solves in a very weak sense:

102 $$\begin{aligned} \int _{\textsf{D}}\beta _{\Omega }\nabla U_0 \cdot \nabla \varphi \;dx =&\textrm{sgn}_{\Omega }(\{x_0\}) \frac{\beta _2-\beta _1}{|\omega |}\int _{\omega } (\nabla K + \nabla u_0(x_0))\cdot \nabla \varphi (x_0) \;dx, \end{aligned}$$

for all $\varphi \in C^1_c({\textsf{D}})$. In fact, $\xi _{x_0}\cdot \nabla E(x-x_0)\in L^q({\textsf{D}})$ for $q\in [1,\frac{d}{d-1})$ and thus one readily verifies that $U_0(x)$ is indeed a very weak solution as defined in Definition 84.

With this we can state our next main result linking the asymptotic expansion and the topological state derivative:

#### Theorem 3.6

For $x_0\in {\textsf{D}}\setminus \partial \Omega$ and $\omega \subset {\textbf{R}}^d$ with $0\in \omega$ we use the definition of $\Omega _{\varepsilon }(x_0,\omega )$ of (11) and set $u_{\varepsilon }:= u_{\Omega _{\varepsilon }(x_0,\omega )}$ and $u_0:= u_\Omega$ and

103 $$\begin{aligned} U_{\varepsilon }:= \frac{u_{\varepsilon }- u_0}{|\omega _{\varepsilon }|}, \quad {\varepsilon }>0. \end{aligned}$$

Let *K* and *v* be defined by (93) and (95), respectively. Then we have the following results.

(i) The asymptotic expansion (96) yields that there is a constant $C=C_{q,d}>0$, which depends on *q* and *d*, such that for all $q\in (1,\frac{d}{d-1})$
  104 $$\begin{aligned} \Vert U_{\varepsilon }-|\omega |^{-1}(R(x-x_0)+v)\Vert _{L^q({\textsf{D}})}\le C {\varepsilon }^{\frac{d-q(d-1)}{q}}\quad \text { for all } {\varepsilon }>0. \end{aligned}$$
(ii) For a.e. $$x\in {\textsf{D}}$$:
  105 $$\begin{aligned} U_0(x) = \lim _{{\varepsilon }\searrow 0}\frac{1}{|\omega _{\varepsilon }|}( {\varepsilon }K(T^{-1}_{\varepsilon }(x)) + {\varepsilon }^d v(x)), \end{aligned}$$
  and thus in particular
  106 $$\begin{aligned} U_0(x) = |\omega |^{-1}(\xi _{x_0}\cdot \nabla E(x-x_0) + v(x)), \end{aligned}$$
  with
  107 $$\begin{aligned} \xi _{x_0} := \textrm{sgn}_{\Omega }(\{x_0\})\frac{\beta _2-\beta _1}{\beta (x_0)}\left( \int _\omega \nabla u_0(x_0)\; dy + \int _\omega \nabla K(y) \;dy \right) . \end{aligned}$$

Note that for $q\in (1,\frac{d}{d-1})$, the exponent ${\frac{d-q(d-1)}{q}}$ is indeed positive.

#### Remark 3.7

In (i) we only claim the convergence rate ${\frac{d-q(d-1)}{q}}$ for $q>1$ while $q=1$, which would correspond to the convergence rate ${\varepsilon }$, is excluded. Obviously we also obtain strong convergence in $L^1$ via Hölder’s inequality, but the estimate (104) only holds for $q\in (1,\frac{d}{d-1})$.

## Topological Derivatives of Shape Functions Via Topological State Derivative

### Topological Differentiability of Shape Functionals

***Semilinear Problem***

We denote by $S(\Omega )=u_\Omega$ the solution operator of the semilinear equation (24). We now discuss the differentiability of $\Omega \mapsto J(\Omega ) = {G}(S(\Omega ))$ for a cost functional ${G}:W^{1,q}({\textsf{D}})\rightarrow {\textbf{R}}$, $q\in [1,\frac{d}{d-1})$. In fact, under sufficient differentiability assumptions on *J* and if ${G}'(u_\Omega ):W^{1,q^\prime }({\textsf{D}}) \rightarrow {\textbf{R}}$ is well-defined, we can show that

$$\begin{aligned} DJ(\Omega )(E) = \lim _{{\varepsilon }\searrow 0} \frac{{G}(u_{{\Omega }(E_{\varepsilon })}) - {G}(u_{\Omega })}{|E_{\varepsilon }|} = {G}'(S(\Omega ))(S'(\Omega )(E)), \end{aligned}$$

where $E\subset {\Omega }$ or $E\subset {\textsf{D}}\backslash {\Omega }$ is either a point, $d-1$-dimensional Lipschitz surface or $1<k<d-1$ dimensional compact and smooth submanifold and $\Omega (E_{\varepsilon })$ is defined in (1.1). Our goal is now to compute the limit ${\varepsilon }\searrow 0$ of

$$\begin{aligned} U_{\varepsilon }:=\frac{u_{\varepsilon }-u_0}{|E_{\varepsilon }|}, \quad {\varepsilon }>0, \end{aligned}$$

where $u_{\Omega }$ is the solution to the semilinear problem (24). To simplify notation, we define again for every ${\varepsilon }>0$ the functions $u_{\varepsilon }:= u_{{\Omega }(E_{\varepsilon })}$, $u_0:=u_{\Omega }$. Recall by Theorem 2.7, $U_{\varepsilon }\rightarrow U_{0}=S'(\Omega )(E)$ in $L^q({\textsf{D}})$, $q\in [1,\frac{d}{d-1})$. Moreover we have according to [9, Lemma 4.4] that $u_{\varepsilon }\rightarrow u_0=u_{\Omega }$ in $H^1_0({\textsf{D}})$ and thus via the Sobolev inequality $u_{\varepsilon }\rightarrow u_0$ in $L^p({\textsf{D}})$ for $1\le p<\frac{2d}{d-2}$ for $d\ge 3$ and $1\le p<\infty$ for $d=2$.

#### Example 4.1

($L^2$ tracking-type) A classical example of a cost functional ${G}(\cdot )$ is of tracking-type, that is:

108 $$\begin{aligned} {G}(u):= \int _{\textsf{D}}(u -u_{\text {ref}})^2 \;dx, \quad u_{\text {ref}}\in L^2({\textsf{D}}). \end{aligned}$$

Consequently, the topological derivative of $J(\Omega ):= {G}(u_\Omega )$ is given by

109 $$\begin{aligned} DJ(\Omega )(E) = \lim _{{\varepsilon }\searrow 0}\int _{\textsf{D}}U_{{\varepsilon }}(u_{\varepsilon }+u_0-2u_{\text {ref}})\;dx = 2\int _{\textsf{D}}S'(\Omega )(E)(u_\Omega - u_{\text {ref}})\;dx, \end{aligned}$$

#### Example 4.2

($L^r$ tracking) More generally we can differentiate for $r>2$

110 $$\begin{aligned} {G}(u):= \int _{\textsf{D}}(u-u_{\text {ref}})^r\;dx, \quad u_{\text {ref}}\in L^\infty ({\textsf{D}})k. \end{aligned}$$

Let again $J(\Omega ):= {G}(u_\Omega )$. Then we have

111 $$\begin{aligned} DJ(\Omega )(E) = r\int _{\textsf{D}}S'(\Omega )(E)(u_\Omega -u_{\text {ref}})^{r-1}\;dx. \end{aligned}$$

Another classical example is the gradient tracking type cost functional.

#### Example 4.3

($L^2$ gradient tracking) For $u_{\text {ref}}\in W^{1,q^\prime }({\textsf{D}})$, consider the gradient-tracking functional

112 $$\begin{aligned} {G}(u):= \int _{\textsf{D}}|\nabla u -\nabla u_{\text {ref}}|^2 \;dx. \end{aligned}$$

A similar computation to the previous one shows

113 $$\begin{aligned} DJ(\Omega )(E)=2\int _{\textsf{D}}\nabla \left( S'(\Omega )(E)\right) \cdot \nabla (u_\Omega - u_{\text {ref}})\;dx. \end{aligned}$$

***Transmission Problem***

Note that due to the weaker convergence result for the transmission problem, the computation of the topological derivative can be more involved for certain cost functionals. We give the following examples.

Let $\Omega \in {{\mathcal {A}}}({\textsf{D}})$. For $x_0\in {\textsf{D}}{\setminus } \partial \Omega$ and a simply connected $\omega \subset {\textbf{R}}^d$ with $0\in \omega$ we use the definition of $\Omega _{\varepsilon }(x_0,\omega )$ given in (11). We denote by $u_{\Omega }$ the solution to (74). We set $u_{\varepsilon }:= u_{\Omega _{\varepsilon }(x_0,\omega )}$ and $u_0:= u_\Omega$ and

114 $$\begin{aligned} U_{\varepsilon }:= \frac{u_{\varepsilon }- u_0}{|\omega _{\varepsilon }|}, \quad {\varepsilon }>0. \end{aligned}$$

Recall that according to Theorem 3.3 we have $U_{\varepsilon }\rightarrow U_0$ as ${\varepsilon }\searrow 0$ in $L^q({\textsf{D}})$ for $q\in [1,\frac{d}{d-1})$. We also have according to [9, Lemma 4.4] that $u_{\varepsilon }\rightarrow u_0$ in $H^1_0({\textsf{D}})$, which implies by the Sobolev inequality $u_{\varepsilon }\rightarrow u_0$ in $L^p({\textsf{D}})$ for $1\le p<\frac{2d}{d-2}$ for $d\ge 3$ and $1\le p<\infty$ for $p=2$. With the definition of $\Omega _{\varepsilon }(x_0,\omega )$ given in (76), we define the topological derivative for $x_0\in {\textsf{D}}{\setminus }\partial \Omega$:

115 $$\begin{aligned} DJ(\Omega )(x_0,\omega ) = \lim _{{\varepsilon }\searrow 0} \frac{J(\Omega _{\varepsilon }(x_0,\omega )) - J(\Omega )}{|\omega _{\varepsilon }|}. \end{aligned}$$

Notice that in case $\omega =B_1(0)$ we have with $E=\{x_0\}$ that $DJ(\Omega )(E) = DJ(\Omega )(x_0,\omega )$, so the previous definition of the topological derivative given in (2) coincides with (115). In contrast to the semilinear problem the topological derivative for the transmission problem actually depends on the shape $\omega$ of the inclusion.

#### Example 4.4

($L_2$ tracking-type) For $u_{\text {ref}}\in L^{q^\prime }({\textsf{D}})$ consider the tracking type cost functional

116 $$\begin{aligned} J(u_\Omega ):= \int _{\textsf{D}}(u_\Omega -u_{\text {ref}})^2 \;dx. \end{aligned}$$

The topological derivative is given by

117 $$\begin{aligned} DJ(\Omega )(x_0,\omega ) = \lim _{{\varepsilon }\searrow 0}\int _{\textsf{D}}U_{{\varepsilon }}(u_{\varepsilon }+u_0-2u_{\text {ref}})\;dx = 2\int _{\textsf{D}}U_0(u_\Omega - u_{\text {ref}})\;dx. \end{aligned}$$

In view of the regularity of $U_0$ and $u_{\Omega },u_{\text {ref}}$ the last integral is indeed well-defined.

As a second example we consider the energy minimisation, where the topological derivative cannot be directly computed via the topological state derivative.

#### Example 4.5

($L_2$ gradient tracking) Let

118 $$\begin{aligned} J(u_\Omega ):= \int _{\textsf{D}}\beta _\Omega |\nabla u_\Omega |^2 \;dx. \end{aligned}$$

As before, we compute

119 $$\begin{aligned} DJ(\Omega )(x_0,\omega ) = \lim _{{\varepsilon }\searrow 0}\int _{\textsf{D}}\beta _\Omega \nabla U_{\varepsilon }\cdot (\nabla u_{\varepsilon }+\nabla u_0)\;dx+\frac{1}{|\omega _{\varepsilon }|}\int _{\omega _{\varepsilon }}(\beta _1-\beta _2)|\nabla u_{\varepsilon }|^2\;dx. \end{aligned}$$

Since the convergence $\nabla U_{\varepsilon }\rightarrow \nabla U_0$ as ${\varepsilon }\searrow 0$ does not hold in $L^p({\textsf{D}})^d$ for $p\ge 2$, we cannot pass to the limit. However, it can be shown using a Lagrangian framework as in [9, 11] that, in fact, the first derivative (119) exists. Thus, this example shows a clear limitation of the topological state derivative. Even though the limit in (119) exists, this method, which relies on the chain rule, is not able to compute the first order topological derivative.

### Expression of Topological Derivative of Functionals with Adjoint Equation

***Semilinear Problem***

We now express the derivative of $J(\Omega ) = G(u_\Omega )$, where $G:W^{1,q^\prime }({\textsf{D}})\rightarrow {\textbf{R}}$, with $q'>d$ (or equivalently $1\le q<\frac{d}{d-1}$), is given, for the semilinear problem in terms of the adjoint equation. We introduce the adjoint state $p_{\Omega }\in W^{1,q^\prime }_0({\textsf{D}})$, that is, the unique solution of120

#### Theorem 4.6

Assume that ${G}(\cdot )$ is differentiable, such that with $J(\Omega ):= {G}(u_\Omega )$ it holds

121 $$\begin{aligned} DJ(\Omega )(E) = {G}'(S(\Omega ))(S'(\Omega )(E)). \end{aligned}$$

Then we have with $W_{\Omega }= \left\{ (g_2(u_{\Omega })-g_1(u_{\Omega }))+(f_1-f_2)\right\} p_{\Omega }$:

122 $$\begin{aligned} DJ({\Omega })(E) = -\textrm{sgn}_{\Omega }(E) \mu _{E}(W_{\Omega }). \end{aligned}$$

- For $E=\{x_0\}$ and $x_0\in {\textsf{D}}\setminus \partial {\Omega }$ we have $\mu _{E}(W_{\Omega }) = W_{\Omega }(x_0)$.
- For $E=\Gamma$ for $\Gamma \subset {\textsf{D}}\setminus \partial {\Omega }$, where $\Gamma$ is a smooth hypersurface of ${\textbf{R}}^d$, we have
  123 $$\begin{aligned} \mu _{E}(W_{\Omega }) = \frac{1}{\text {Per}(\Gamma )}\int _{\Gamma } W_{\Omega }\; d{{\mathcal {H}}}^{d-1}. \end{aligned}$$
- For $E=M$ for $M\subset {\textsf{D}}\setminus \partial \Omega$, where *M* is a smooth *k*-submanifold, $1< k <d-1$ of ${\textbf{R}}^d$ without boundary, we have
  124 $$\begin{aligned} \mu _{E}(W_{\Omega }) = \frac{1}{{{\mathcal {H}}}^k(M)}\int _{M} W_\Omega \; d{{\mathcal {H}}}^k. \end{aligned}$$

#### Proof

Recalling that by definition $S({\Omega })=u_{\Omega }$, we have

$$\begin{aligned} DJ({\Omega })(E)&= {G}'(S({\Omega }))(S'({\Omega })(E)) \\&{\mathop {=}\limits ^{(120)}} - \int _{\textsf{D}}\nabla p_{\Omega }\cdot \nabla S'({\Omega })(E) + \partial _u\rho _{\Omega }(x,u_{\Omega })p_{\Omega }S'({\Omega })(E) \;dx \\&{\mathop {=}\limits ^{(29)}} -\textrm{sgn}_{\Omega }(E)\mu _{E}(W_\Omega ). \end{aligned}$$

This concludes the proof. $\square$

#### Example 4.7

Consider $J(\Omega )={G}(u_\Omega )$ and again the tracking-type cost functional of Example 4.1, namely,

$$\begin{aligned} {G}(u) = \int _{\textsf{D}}(u - u_{\text {ref}})^2\;dx. \end{aligned}$$

The adjoint state is the (unique) solution $p_{\Omega }\in W^{1,q^\prime }_0(\Omega )$, $q\in [1,\frac{d}{d-1})$, of125and thus the topological derivative of the shape functional reads:

126 $$\begin{aligned} DJ({\Omega })(E) = \textrm{sgn}_{\Omega }(E) \mu _{E}(\left\{ (g_2(u_{\Omega })-g_1(u_{\Omega }))+(f_1-f_2)\right\} p_{\Omega }). \end{aligned}$$

We finish with the gradient-tracking example.

#### Example 4.8

Consider $J(\Omega ) = {G}(u_\Omega )$ with the gradient-tracking function ${G}(\cdot )$ of Example 4.3:

127 $$\begin{aligned} {G}(u)= \int _{\textsf{D}}|\nabla u -\nabla u_{\text {ref}}|^2 \;dx. \end{aligned}$$

The adjoint state is the (unique) solution $p_{\Omega }\in W^{1,q^\prime }_0(\Omega )$, $\in [1,\frac{d}{d-1})$, of128and thus the derivative is again given by

129 $$\begin{aligned} DJ({\Omega })(E) = \textrm{sgn}_{\Omega }(E) \mu _{E}(\left\{ (g_2(u_{\Omega })-g_1(u_{\Omega }))+(f_1-f_2)\right\} p_{\Omega }). \end{aligned}$$

***Transmission Problem***

We consider the transmission problem and cost function $J(\Omega ) = G(u_\Omega )$ with *G* defined in Example 4.4:

130 $$\begin{aligned} G(u_\Omega ):= \int _{\textsf{D}}(u_\Omega -u_{\text {ref}})^2 \;dx. \end{aligned}$$

We derived the following form of the topological derivative for $x_0\in {\textsf{D}}\setminus \partial \Omega$:

131 $$\begin{aligned} DJ(\Omega )(x_0,\omega ) = 2\int _{\textsf{D}}U_0(u_\Omega - u_{\text {ref}})\;dx. \end{aligned}$$

Now introduce the adjoint associated with the cost functional (130), namely, $p_\Omega \in H^1_0({\textsf{D}})$ that solves in a weak sense $-{\text {div}}(\beta _\Omega \nabla p_\Omega ) = -2(u_\Omega -u_{\text {ref}})$ in ${\textsf{D}}$. By (86) we know that $U_0$ solves

132 $$\begin{aligned} \int _{\textsf{D}}U_0 v \;dx = \textrm{sgn}_{\Omega }(\{x_0\}) (\beta _2-\beta _1)\left( \frac{1}{|\omega |}\int _\omega \nabla K + \nabla u_\Omega (x_0)\; dx\right) \cdot \nabla \varphi _v(x_0) \end{aligned}$$

for all $v\in L^{q^\prime }({\textsf{D}})$, where $\varphi _v$ is the solution to (79). By definition of $\varphi _v$ we readily verify that $p_\Omega = \varphi _v$ for $v:= -2(u_\Omega -u_{\text {ref}})$. Therefore testing (132) with $v=-2(u_\Omega -u_{\text {ref}})$ yields together with (131):

133 $$\begin{aligned} DJ(\Omega )(x_0,\omega ) = -\textrm{sgn}_{\Omega }(\{x_0\}) (\beta _2-\beta _1)\left( \frac{1}{|\omega |}\int _\omega \nabla K + \nabla u_\Omega (x_0)\; dx\right) \cdot \nabla p_\Omega (x_0). \end{aligned}$$

In case $\omega =B_1(0)$, using Remark 3.4, we can express the topological derivative of *J* by

134 $$\begin{aligned} DJ(\Omega )(x_0,\omega ) = -\textrm{sgn}_{\Omega }(\{x_0\}) (\beta _2-\beta _1)(C_{\beta }+1) \nabla u_\Omega (x_0)\cdot \nabla p_\Omega (x_0). \end{aligned}$$

## Proofs for Semilinear Problems

### Preliminary Results on Bilinear Elliptic Equations with Measure Data

In this first section we give the basic regularity estimates for bilinear elliptic equations with measure data; the following proposition will be useful when considering the well-posedness of linearised systems. It should be noted that this result is not an immediate consequence of [22] or [34] but that the methods used to derive it is inspired by these contributions.

#### Proposition 5.1

Let $\Psi \in L^\infty ({\textsf{D}})$ be such that the first eigenvalue $\lambda _1(\Psi )$ of the operator $L_\Psi :=-\Delta +\Psi$ is positive:

$$\begin{aligned} \lambda _1(\Psi )=\inf _{u\in H^{1}_0({\textsf{D}})\backslash \{0\}} \frac{\int _{\textsf{D}}|\nabla u|^2\;dx+\int _{\textsf{D}}\Psi u^2\;dx}{\int _{\textsf{D}}u^2\;dx}>0. \end{aligned}$$

Then, for every $\mu \in {\mathcal {M}}({\textsf{D}})$ there exists a unique *u* that satisfies135in the weak $W^{1,q}_0({\textsf{D}})$-sense for every $q\in [1,\frac{d}{d-1})$. Furthermore, for every $q\in [1,\frac{d}{d-1})$, there exists a constant $C_q$ such that, for every $\mu \in {\mathcal {M}}({\textsf{D}})$,

$$\begin{aligned} \Vert u\Vert _{W^{1,q}({\textsf{D}})}\le C_q \Vert \mu \Vert _{{\mathcal {M}}({\textsf{D}})}. \end{aligned}$$

#### Proof of Proposition 5.1

$\underline{\textrm{Approximation}\,\textrm{of}\,\mathrm{(135)}:}$ We follow a standard [22, Lemma 3.4] approximation scheme: for every $\mu \in {\mathcal {M}}({\textsf{D}})$ we consider a sequence $\{\mu _k\}_{k\in {\textbf{N}}}$ of $C^\infty$ functions that converges weakly (in the sense of measures) to $\mu$ and such that

136 $$\begin{aligned} \lim _{k\rightarrow \infty }\Vert \mu _k\Vert _{L^1({\textsf{D}})}=\Vert \mu \Vert _{{\mathcal {M}}({\textsf{D}})}.\end{aligned}$$

We consider the system (27) with $\mu$ replaced with $\mu _k$:137The existence of a solution $v_k$ to (137) follows from the minimisation of the energy functional

$$\begin{aligned} {\mathscr {E}}_k:H^{1}_0({\textsf{D}})\ni u\mapsto \frac{1}{2}\int _{\textsf{D}}|\nabla u|^2\;dx+\frac{1}{2}\int _{\textsf{D}}\Psi u^2\;dx-\int _{\textsf{D}}\mu _k u\;dx.\end{aligned}$$

To check that ${\mathscr {E}}_k$ is indeed coercive, we use the fact that $\lambda _1(\Psi )>0$ to obtain: for every $u\in H^{1}_0({\textsf{D}})$,

$$\begin{aligned} {\mathscr {E}}_k(u)\ge \frac{\lambda _1(\Psi )}{2}\int _{\textsf{D}}u^2\;dx-\int _{\textsf{D}}\mu _ku\;dx. \end{aligned}$$

Regarding the uniqueness, observe that if there are two different solutions $(v_k,v_k')$ of (137) then the difference $z_k:=v_k-v_k'$ and satisfies138Multiplying (138) by $z_k$ and integrating by parts we obtain

$$\begin{aligned} \int _{\textsf{D}}|\nabla z_k|^2\;dx+\int _{\textsf{D}}\Psi z_k^2\;dx=0. \end{aligned}$$

Since $\lambda _1(\Psi )>0$, this is implies $z_k=0$, which means $v_k=v_k'$ and hence shows uniqueness.

$\underline{\text {Regularity estimates on the approximated problem:}}$ In order to derive $W^{1,p}$-estimates on the sequence $\{v_k\}_{k\in {\textbf{N}}}$, we begin with an *a priori* $L^1$-estimate on $\{v_k\}_{k\in {\textbf{N}}}$; it will then suffice to apply the classical regularity result [22, Proposition 4.1]. Consider, for every function $h\in L^\infty ({\textsf{D}})$, the solution $\theta _h$ of139The existence and uniqueness of a solution to (139) follows from the same energy argument already used to obtain existence and uniqueness for $v_k$. We claim that for every $p\in [2,\infty )$, there exists a constant $C_p$ such that

140 $$\begin{aligned} \Vert \theta _h\Vert _{W^{2,p}({\textsf{D}})}\le C_p\Vert h\Vert _{L^p({\textsf{D}})}. \end{aligned}$$

(140) follows from a standard bootstrap argument which we just show the initialisation of. Using $\theta _h$ as a test function in the weak formulation of (139) we obtain

$$\begin{aligned} \int _{\textsf{D}}\theta _h^2\;dx\le \frac{1}{\lambda _1(\Psi )}\int _{\textsf{D}}h^2\;dx, \end{aligned}$$

whence elliptic regularity guarantees $\Vert \theta _h\Vert _{W^{2,2}({\textsf{D}})}\le C \Vert h\Vert _{L^2({\textsf{D}})}$. This implies (140) for $p=2$. Now using the Sobolev embedding $W^{2,2}({\textsf{D}})\hookrightarrow L^p({\textsf{D}})$ ($p>2$) and writing $-\Delta \theta _h = h-\Psi \theta _h\in L^p({\textsf{D}})$, $p>2$, we conclude again by elliptic regularity that $\theta _h\in W^{2,p}({\textsf{D}})$ and

$$\begin{aligned} \Vert \theta _h\Vert _{W^{2,p}({\textsf{D}})}\le C(\Vert \theta _h\Vert _{L^p({\textsf{D}})}+ \Vert h\Vert _{L^p({\textsf{D}})}) \le C(\Vert \theta _h\Vert _{W^{2,2}({\textsf{D}})} + \Vert h\Vert _{L^p({\textsf{D}})}) \le C\Vert h\Vert _{L^p({\textsf{D}})}, \end{aligned}$$

which is (140). Consequently, there exists a constant *C*, such that

$$\begin{aligned} \Vert \theta _h\Vert _{L^\infty ({\textsf{D}})}\le C \Vert h\Vert _{L^\infty ({\textsf{D}})}. \end{aligned}$$

Now, use $\theta _h$ as a test function in (137). We obtain, integrating by parts twice,

$$\begin{aligned} \left| \int _{\textsf{D}}v_k h\;dx\right| \le \left| \int _{\textsf{D}}\theta _h \mu _k\;dx\right| \le C \Vert \mu _k\Vert _{L^1({\textsf{D}})}\Vert h\Vert _{L^\infty ({\textsf{D}})}. \end{aligned}$$

By (136) we deduce that there exists a constant *C* such that $\sup _{k\in {\textbf{N}}}\Vert v_k\Vert _{L^1({\textsf{D}})}\le C\Vert \mu \Vert _{{\mathcal {M}}({\textsf{D}})}.$ Observe now that we can rewrite the equation on $v_k$ as $-\Delta v_k={\tilde{\mu }}_k$ with ${\tilde{\mu }}_k=\mu _k-\Psi v_k.$ As $\Psi \in L^\infty ({\textsf{D}})$ we have, for a certain constant *C*, that $\Vert {\tilde{\mu }}_k\Vert _{{\mathcal {M}}({\textsf{D}})}\le C \Vert \mu \Vert _{{\mathcal {M}}({\textsf{D}})}$. Consequently, from [22, Proposition 4.1] we know that, for every $q\in [1,\frac{d}{d-1})$, there exists a constant $c_q$ such that, for every $k\in {\textbf{N}}$,

$$\begin{aligned} \Vert v_k\Vert _{W^{1,q}({\textsf{D}})}\le c_q \Vert \mu \Vert _{{\mathcal {M}}({\textsf{D}})}. \end{aligned}$$

We can thus extract a $W^{1,q}({\textsf{D}})$-weak, $L^q({\textsf{D}})$-strong, converging subsequence of $\{v_k\}_{k\in {\textbf{N}}}$. Let *U* be the closure point under consideration. For every $p>d$, Sobolev embeddings imply that $W^{1,p}({\textsf{D}})\hookrightarrow C^0({\overline{{\textsf{D}}}})$. Let $\theta \in W^{1,p}({\textsf{D}})$. Passing to the limit in the identity

$$\begin{aligned} \int _{\textsf{D}}\nabla v_k\cdot \nabla \theta \;dx+\int _{\textsf{D}}\Psi v_k \theta \;dx=\int _{\textsf{D}}\theta \mu _k\;dx \end{aligned}$$

it appears that *U* solves (27) in the weak $W^{1,q}$-sense, and further satisfies the required regularity estimate. The existence of a solution is thus established.

$\underline{\text {Uniqueness of a solution to (27):}}$ Assume that $u_1,u_1$ are two distinct solutions of (27). Then $z:=u_1-u_2$ satisfies141It is then clear that *z* solves (141) in the weak $H^{1}_0$-sense. However, we already proved (see above the proof of $z_k\equiv 0$) that this implies $z=0$ and thus the uniqueness. Thus the solution *u* is necessarily unique. $\square$

In this section we gather the proofs of Proposition 2.6, Theorems 2.7, 2.8, 2.10 and Corollary 2.11.

### Proof of Proposition 2.6

It suffices to prove that the potential

142 $$\begin{aligned} W_{\Omega }:=\frac{\partial \rho _{\Omega }}{\partial u}(x,u_{\Omega }) \end{aligned}$$

is such that the assumptions of Proposition 5.1 are satisfied. Given that Assumption (23) is satisfied, we know that

143 $$\begin{aligned} W_{\Omega }\ge 0. \end{aligned}$$

Furthermore, by standard elliptic regularity, $u_{\Omega }\in L^\infty ({\textsf{D}})$, whence we conclude $W_{\Omega }\in L^\infty ({\textsf{D}}).$ Consequently, the first eigenvalue $\lambda _1(W_{\Omega })$ (with the notations of Proposition 5.1) is bounded from below:

$$\begin{aligned} \lambda _1(W_{\Omega })\ge \inf _{\begin{array}{c} u\in H^{1}_0({\textsf{D}}) \\ u \ne 0 \end{array}} \frac{\int _{\textsf{D}}|\nabla u|^2\;dx}{\int _{\textsf{D}}u^2\;dx} > 0, \end{aligned}$$

where the infimum on the right hand side is the first Dirichlet eigenvalue of the domain ${\textsf{D}}$. It suffices to use Proposition 5.1 to obtain the conclusion.

### Proof of Theorem 2.7

The computations are similar whether we take $E\subset {\Omega }$ or $E\subset {\textsf{D}}\backslash {\overline{{\Omega }}}$. Thus, for notational simplicity, we consider the case $E\subset {\textsf{D}}\backslash {\overline{{\Omega }}}$. The function $U_{\varepsilon }$ solves: $U_{\varepsilon }=0$ on $\partial {\textsf{D}}$ and in a weak $W^{1,q}_0({\textsf{D}})$ sense:

$$\begin{aligned} -\Delta U_{\varepsilon }+&\chi _{\Omega }\frac{(g_1(u_{\varepsilon })-g_1(u_0)-g_2(u_{\varepsilon })+g_2(u_0))}{|E_{\varepsilon }|} \\&+\mu _{\varepsilon }(g_1(u_{\varepsilon })-g_2(u_{\varepsilon }))+\frac{g_2(u_{\varepsilon })-g_2(u_0)}{|E_{\varepsilon }|} =(f_1-f_2)\mu _{\varepsilon }. \end{aligned}$$

Now observe that, as $\chi _{{\Omega }_{\varepsilon }(E)}\underset{{\varepsilon }\searrow 0}{\rightarrow }\chi _{\Omega }$ in $L^2({\textsf{D}})$, elliptic regularity estimates entail

144 $$\begin{aligned} u_{\varepsilon }\underset{{\varepsilon }\searrow 0}{\rightarrow }u_0\text { in } C^0({\overline{{\textsf{D}}}}). \end{aligned}$$

By the mean value theorem, for $i=1,2$ and $x\in {\textsf{D}}$, there exists $s_{{\varepsilon },i}(x)\in [0,1]$ such that $v_{{\varepsilon },i}(x):=s_{{\varepsilon },i}(x)u_{\varepsilon }(x)+\left( 1-s_{{\varepsilon },i}(x)\right) u_0(x)$ satisfies

$$\begin{aligned} g_i(u_{\varepsilon })-g_i(u_0)=-g_i'(v_{{\varepsilon },i})(u_{\varepsilon }-u_0)\quad (i=1,2). \end{aligned}$$

From (144) we have $v_{{\varepsilon },i}\underset{{\varepsilon }\searrow 0}{\rightarrow }u_0$ in $C^0({\overline{{\textsf{D}}}})$. This allows to rewrite the equation on $U_{\varepsilon }$ as

145 $$\begin{aligned} -\Delta U_{\varepsilon }+\chi _{\Omega }{g_1'(v_{{\varepsilon },1})U_{\varepsilon }+\chi _{{\textsf{D}}\backslash {\Omega }}g_2'(v_{{\varepsilon },2})}U_{\varepsilon }=-\mu _{\varepsilon }(g_1(u_{\varepsilon })-g_2(u_{\varepsilon }))+(f_1-f_2)\mu _{\varepsilon }\end{aligned}$$

in ${\textsf{D}}$. From the same regularity estimates derived in the proof of Proposition 2.6 we deduce that, for every $q\in [1,\frac{d}{d-1})$ and $\delta >0$ small,

$$\begin{aligned} \sup _{{\varepsilon }\in (0,\delta ] }\Vert U_{\varepsilon }\Vert _{W^{1,q}({\textsf{D}})}<\infty . \end{aligned}$$

We may thus pass to the weak $W^{1,q}$, strong $L^q$ limit in the equation of $U_{\varepsilon }$ to obtain that every accumulation point of this sequence is a weak $W^{1,q}$-solution to (29). Since the uniqueness of a solution to this equation was established in Proposition 2.6 the conclusion follows, if we can prove that the convergence is, in fact, strong in $W^{1,q}({\textsf{D}})$. Here, we use [30, Assertion (21)] (see also [22, Proposition 4.9]). Rewrite the equation (145) on $U_{\varepsilon }$ as

$$\begin{aligned} -\Delta U_{\varepsilon }=(f_1-f_2-g_1(u_{\varepsilon })+g_2(u_{\varepsilon }))\mu _{\varepsilon }-(\chi _{\Omega }{g_1'(v_{{\varepsilon },1})+\chi _{{\textsf{D}}\backslash {\Omega }}g_2'(v_{{\varepsilon },2})})U_{\varepsilon }={\tilde{\mu }}_{\varepsilon }. \end{aligned}$$

From the strong convergence of $U_{\varepsilon }$ in $L^1({\textsf{D}})$, we deduce from [30, Assertion (21)] that $\{U_{\varepsilon }\}_{{\varepsilon }\in (0,\delta ]}$ is a sequentially compact family in $W^{1,q}_0({\textsf{D}})$ and thus converges strongly. The proof of the proposition is complete.

### Proof of Theorem 2.8

It will be convenient to observe that when $E=\{x_0\}$ the function $U_0$ solves the equation146in a weak $W^{1,q}_0({\textsf{D}})$-sense. The function *F* in (146) is defined as

$$\begin{aligned} F=g_2(u_{\Omega })-g_1(u_{\Omega })+f_1-f_2\in L^\infty ({\textsf{D}}), \end{aligned}$$

and the potential $W_{\Omega }$ is defined in (142). Introduce the Green kernel $G=G_{\Omega }=G_{\Omega }(x,y)$ of the operator $-\Delta +W_{\Omega }$, that is, the unique solution of147A detailed study of the Green kernel of operators *L* having the form $-\sum _i\partial _i(\sum _j a_{i,j}\partial _j)$ can be found in the seminal work [23]. Here, the existence of a Green kernel follows from Proposition 5.1, and the Green kernel is symmetric in the sense that $G(x,y)=G(y,x)$ for all for all $x\ne y\in {\textsf{D}}$. Furthermore, from Proposition 5.1 and Sobolev embeddings we know that,

148 $$\begin{aligned} \forall y\in {\textsf{D}}\,, G(y,\cdot )\in L^2({\textsf{D}}). \end{aligned}$$

Finally, it is clear that for all $y\in {\textsf{D}}\,, U_{\{y\},0}=\textrm{sgn}_{\Omega }(\{y\})F(y) G(y,\cdot )$ and consequently, for every $h\in L^2({\textsf{D}})$, the function

$$\begin{aligned} {\tilde{u}}= x\mapsto \int _{\textsf{D}}\textrm{sgn}_{\Omega }(\{y\}) U_{\{y\},0}(x)h(y)dy=\int _{\textsf{D}}F(y) G(x,y)h(y)dy, \end{aligned}$$

is well-defined. It suffices to differentiate it to obtain that ${\tilde{u}}$ solves (30), thereby concluding the proof of Theorem 2.8.

### Proof of Theorem 2.10

#### Proof

To establish (i) for $d=2$ we have $K(x) = R(x) + O(|x|^{-1})$ as $|x|\rightarrow \infty$ and thus for $x\ne x_0$:

149 $$\begin{aligned} K(T_{\varepsilon }^{-1}(x)) = R(T_{\varepsilon }^{-1}(x)) + O(|T_{\varepsilon }^{-1}(x)|^{-1}). \end{aligned}$$

Since $R(x) = b\ln (|x|)$, it follows $R(T_{\varepsilon }^{-1}(x)) = b\ln (x-x_0) - b\ln ({\varepsilon }) = R(x-x_0) - b\ln ({\varepsilon })$ and thus we conclude for ${\varepsilon }\searrow 0$:

150 $$\begin{aligned} \frac{1}{|\omega _{\varepsilon }|} {\varepsilon }^2(K(T_{\varepsilon }^{-1}(x)) + v(x) + \ln ({\varepsilon })b) = |\omega |^{-1}( b\ln (|x-x_0|) + v(x)) + O(|T_{\varepsilon }^{-1}(x)|^{-1}), \end{aligned}$$

in view of $|T_{\varepsilon }^{-1}(x)|^{-1} = {\varepsilon }|x-x_0|^{-1}$ the result follows. The proof of (48) is established the same way and left to the reader.

For the sake of simplicity we only give a proof for item (iii), that is we restrict ourselves to dimension $d\ge 3$. Note that the same arguments can be used to show the according results of item (ii). We start by proving the last item of (iii). Therefore, first note that we have

$$\begin{aligned} U_{\varepsilon }- |\omega |^{-1}( {\varepsilon }^{2-d}K\circ T_{\varepsilon }^{-1} + v)=|\omega |^{-1}{\varepsilon }^{2-d}\left( K_{\varepsilon }\circ T_{\varepsilon }^{-1}-K\circ T_{\varepsilon }^{-1}-{\varepsilon }^{d-2}v\right) . \end{aligned}$$

Thus, from (44) and changing variables, we obtain

151 $$\begin{aligned} \Vert U_{\varepsilon }- |\omega |^{-1}( {\varepsilon }^{2-d}K\circ T_{\varepsilon }^{-1} + v)\Vert _{H^1({\textsf{D}})} \le C{\varepsilon }. \end{aligned}$$

Now we estimate for $p\in (1,\frac{d}{d-1})$ by the triangle inequality and Hölder’s inequality:

152 $$\begin{aligned} \begin{aligned}&\Vert \nabla (U_{\varepsilon }- |\omega |^{-1}(R(x-x_0)+v))\Vert _{L^p({\textsf{D}})^d} \le \\&\quad C\Vert \nabla (U_{\varepsilon }- |\omega |^{-1}({\varepsilon }^{2-d}K\circ T_{\varepsilon }^{-1}+v))\Vert _{L^2({\textsf{D}})^d} \\&\quad + C|\omega |^{-1}\Vert \nabla ({\varepsilon }^{2-d}K\circ T_{\varepsilon }^{-1} -R(x-x_0))\Vert _{L^p({\textsf{D}})^d}. \end{aligned} \end{aligned}$$

Using the estimate (151), we see that the first term on the right hand side is bounded by $C{\varepsilon }$. For the second term we note $\nabla R({\varepsilon }x) = {\varepsilon }^{1-d}\nabla R(x)$ and thus

$$\begin{aligned} \int _{\textsf{D}}|\nabla ({\varepsilon }^{2-d}K\circ T_{\varepsilon }^{-1} - R(x-x_0))|^p \;dx&= \int _{\textsf{D}}|{\varepsilon }^{1-d} \nabla K(T_{\varepsilon }^{-1}(x)) - \nabla R(x-x_0))^p \;dx \\&= \int _{{\textsf{D}}_{\varepsilon }}{\varepsilon }^d |{\varepsilon }^{1-d}K(x) - \nabla R({\varepsilon }x)|^p \;dx \\&= \int _{{\textsf{D}}_{\varepsilon }}{\varepsilon }^{d-p(d-1)} |K(x) - \nabla R(x)|^p \;dx, \end{aligned}$$

and

153 $$\begin{aligned} \Vert \nabla (K\circ T_{\varepsilon }^{-1} -R(x-x_0))\Vert _{L^p({\textsf{D}})^d}^p = {\varepsilon }^{d-p(d-1)} \Vert \nabla (K-R)\Vert _{L^p({\textsf{D}}_{\varepsilon })^d}^p. \end{aligned}$$

Now according to Lemma 6.1, we have

154 $$\begin{aligned} \Vert \nabla (K-R)\Vert _{L^p({\textsf{D}}_{\varepsilon })^d} \le \Vert \nabla (K-R)\Vert _{L^p({\textbf{R}}^d)^d}<\infty \quad \text { for } p \in \left( 1,\frac{d}{d-1}\right) . \end{aligned}$$

Therefore we obtain for $p\in (1,\frac{d}{d-1})$ noticing that $0<p^{-1}(d-p(d-1))<1$:

155 $$\begin{aligned} \Vert \nabla (U_{\varepsilon }- |\omega |^{-1}(R(x-x_0)+v))\Vert _{L^p({\textsf{D}})^d} \le C{\varepsilon }+ C{\varepsilon }^{\frac{d-p(d-1)}{p}} \le C{\varepsilon }^{\frac{d-p(d-1)}{p}}. \end{aligned}$$

Since by (41), $v(x)=-R(x-x_0)$ and $u_{\varepsilon }(x)=u_0(x)=0$ for $x\in \partial {\textsf{D}}$, we have

$$\begin{aligned} U_{\varepsilon }- |\omega |^{-1}(R(x-x_0)+v) = |\omega |^{-1}(R(x-x_0)-R(x-x_0)) =0 \quad \text { on } \partial {\textsf{D}}. \end{aligned}$$

Therefore the Poincaré inequality yields

$$\begin{aligned} C\Vert U_{\varepsilon }- |\omega |^{-1}(R(x-x_0)+v)\Vert _{L^p({\textsf{D}})} \le \Vert \nabla (U_{\varepsilon }- |\omega |^{-1}(R(x-x_0)+v))\Vert _{L^p({\textsf{D}})^d}, \end{aligned}$$

and hence the last item of (iii) follows.

We now prove the first item in (iii). We compute for $d\ge 3$ and $p\in (\frac{d}{d-1},\frac{d}{d-2})$, using the continuous embedding $H^1({\textsf{D}}) {\mathop {\hookrightarrow }\limits ^{c}} L^p({\textsf{D}})$ for $p\in [1,\frac{d}{d-2})$:

156 $$\begin{aligned} \begin{aligned} \Vert U_{\varepsilon }- |\omega |^{-1}(R(x-x_0)+v)\Vert _{L^p({\textsf{D}})} \le&C\Vert U_{\varepsilon }- |\omega |^{-1}({\varepsilon }^{2-d}K\circ T_{\varepsilon }^{-1}+v)\Vert _{L^p({\textsf{D}})} \\&+ C|\omega |^{-1}\Vert {\varepsilon }^{2-d}K\circ T_{\varepsilon }^{-1}-R(x-x_0)\Vert _{L^p({\textsf{D}})}\\ \le&C\Vert U_{\varepsilon }- |\omega |^{-1}(K\circ T_{\varepsilon }^{-1}+v)\Vert _{H^1({\textsf{D}})} \\&+ C|\omega |^{-1}\Vert {\varepsilon }^{2-d}K\circ T_{\varepsilon }^{-1}-R(x-x_0)\Vert _{L^p({\textsf{D}})}. \end{aligned} \end{aligned}$$

Moreover, we have by changing variables:

157 $$\begin{aligned} \Vert {\varepsilon }^{2-d}K\circ T_{\varepsilon }^{-1}-R(x-x_0)\Vert _{L^p({\textsf{D}})} = {\varepsilon }^{\frac{d-p(d-2)}{p}}\Vert K-R\Vert _{L^p({\textsf{D}}_{\varepsilon })}, \end{aligned}$$

and according to Lemma 6.1

158 $$\begin{aligned} \Vert K-R\Vert _{L^p({\textsf{D}}_{\varepsilon })}\le \Vert K-R\Vert _{L^p({\textbf{R}}^d)}<\infty \quad \text { for }\quad p\in \left( \frac{d}{d-1},\frac{d}{d-2}\right) . \end{aligned}$$

Therefore from (156) and (151), we have for $p\in \left( \frac{d}{d-1},\frac{d}{d-2}\right)$

159 $$\begin{aligned} \Vert U_{\varepsilon }- |\omega |^{-1}(R(x-x_0)+v)\Vert _{L^p({\textsf{D}})} \le C{\varepsilon }+ C{\varepsilon }^{\frac{d-p(d-2)}{p}}\le C{\varepsilon }^{\frac{d-p(d-2)}{p}}. \end{aligned}$$

$$\square $$

### Proof of Corollary 2.11

#### Proof

This is a direct consequence of Theorem 2.10 having a close inspection of the proof of Theorem 2.10, item(ii). Indeed, since $\omega =B_1(0)$, we have according to [26] a.e. in ${\textsf{D}}$:

160 $$\begin{aligned} U_{\varepsilon }- |\omega |^{-1}(K\circ T_{\varepsilon }^{-1}+v +b\ln ({\varepsilon }))=0 \quad \text { for } d=2, \end{aligned}$$

and

161 $$\begin{aligned} U_{\varepsilon }- |\omega |^{-1}({\varepsilon }^{2-d}K\circ T_{\varepsilon }^{-1}+v)=0 \quad \text { for } d\ge 3. \end{aligned}$$

Moreover, $K=R$ on ${\textbf{R}}^d\setminus \overline{B_1(0)}$, that is, the asymptotics of *K* aborts after the first term. Therefore for $d\ge 3$ it follows from (161):

162 $$\begin{aligned} \Vert U_{\varepsilon }- |\omega |^{-1}(R(x-x_0)+v)\Vert _{L^p({\textsf{D}})} \le&C|\omega |^{-1}\Vert {\varepsilon }^{2-d}K\circ T_{\varepsilon }^{-1}-R(x-x_0)\Vert _{L^p(B_{\varepsilon }(0))}. \end{aligned}$$

Now changing variables $T_{\varepsilon }(x)=y$ and $R(T_{\varepsilon }(x)-x_0)={\varepsilon }^{-1}R(x)$ shows that

163 $$\begin{aligned} \Vert {\varepsilon }^{2-d}K\circ T_{\varepsilon }^{-1}-R(x-x_0)\Vert _{L^p(B_{\varepsilon }(0))} = {\varepsilon }^{\frac{d-p(d-2)}{p}}\Vert K-R\Vert _{L_p(B_1(0))} \end{aligned}$$

the last term is finite for $p\in [1,\frac{d}{d-1})$ since $R(x)=E(x)=|\omega |c_d|x|^{-(d-2)}\in L_p(B_1(0))$ for such *p* (recall *E* was defined in (38)). The case $d=2$ is treated in the same fashion noting that for $d=2$ we have $R(x)=-|\omega |(2\pi )^{-1}\ln (|x|)\in L_p(B_1(0))$ for all $p\ge 1$. $\square$

## Proofs for the Transmission Problem

### Proof of Proposition 3.2

$\underline{\text {Existence of a very weak solution}}$ To establish the existence of a solution, it suffices to observe that the linear map

$$\begin{aligned} T:L^p({\textsf{D}})\ni v\mapsto \zeta \cdot \nabla \varphi _v(x_0) \end{aligned}$$

is continuous for every $p>d$, as $\Vert \varphi _v\Vert _{ C^1({\overline{\Omega }}\cup ({\textsf{D}}{\setminus }{\overline{\Omega }}))}\le C \Vert v\Vert _{L^p({\textsf{D}})}$ by elliptic regularity. By the Riesz representation theorem, there exists a unique $\varphi _{\zeta ,x_0}\in L^{p'}({\textsf{D}})$, such that

164 $$\begin{aligned} \int _{\textsf{D}}\varphi _{\zeta ,x_0}v = T(v) \quad \text { for every } v\in L^p({\textsf{D}}). \end{aligned}$$

Therefore (84) admits a unique solution $u\in L^{p'}({\textsf{D}})$, which further satisfies the required regularity estimates.

### Proof of Theorem 3.3

#### Proof of Theorem 3.3

Let $u_{\varepsilon }:= u_{\Omega _{\varepsilon }(x_0,\omega )}$, $u_0:=u_{\Omega }$ and $U_{\varepsilon }= \frac{u_{\varepsilon }-u_0}{|\omega _{\varepsilon }|}$. Then we obtain

165 $$\begin{aligned}&\int _{\textsf{D}}\beta _{\Omega }\nabla (u_{\varepsilon }- u_0)\cdot \nabla \varphi \;dx = \end{aligned}$$

166 $$\begin{aligned}&\textrm{sgn}_{\Omega }(\{x_0\}) (\beta _2-\beta _1)\bigg (\int _{\omega _{\varepsilon }}&\nabla (u_{\varepsilon }-u_0) \cdot \nabla \varphi \;dx + \int _{\omega _{\varepsilon }} \nabla u_0(x)\cdot \nabla \varphi \;dx \bigg ), \end{aligned}$$

for all $\varphi \in H^1_0({\textsf{D}})$. Dividing by $|\omega _{\varepsilon }| = |\omega |{\varepsilon }^d$ and using $K_{\varepsilon }= \frac{(u_{\varepsilon }-u_0)\circ T_{\varepsilon }}{{\varepsilon }}$, this can be written as

$$\begin{aligned} \int _{\textsf{D}}\beta _{\Omega }\nabla U_{\varepsilon }\cdot \nabla \varphi \;dx =&\, \textrm{sgn}_{\Omega }(\{x_0\}) (\beta _2-\beta _1)\frac{1}{|\omega _{\varepsilon }|}\int _{\omega _{\varepsilon }} \nabla K_{\varepsilon }\circ T_{\varepsilon }^{-1} \cdot \nabla \varphi (x) \;dx\\ {}&+ \textrm{sgn}_{\Omega }(\{x_0\}) (\beta _2-\beta _1)\frac{1}{|\omega _{\varepsilon }|}\int _{\omega _{\varepsilon }} \nabla u_0(x)\cdot \nabla \varphi (x) \;dx, \end{aligned}$$

for all $\varphi \in H^1_0({\textsf{D}})$. Now choosing $\varphi = \varphi _v$ (with $\varphi _v$ defined in (79) for $v\in L^p({\textsf{D}})$, $p>d$ and $q:=p'=q/(q-1)$) as a test function and integrating by parts in the first integral using $-{\text {div}}(\beta _\Omega \nabla \varphi _v) = v$ yields the very weak formulation:

$$\begin{aligned} \int _{\textsf{D}}U_{\varepsilon }v \;dx =&\, \textrm{sgn}_{\Omega }(\{x_0\}) (\beta _2-\beta _1)\frac{1}{|\omega _{\varepsilon }|}\int _{\omega _{\varepsilon }} \nabla K_{\varepsilon }\circ T_{\varepsilon }^{-1} \cdot \nabla \varphi _v(x) \;dx\\ {}&+ \textrm{sgn}_{\Omega }(\{x_0\}) (\beta _2-\beta _1)\frac{1}{|\omega _{\varepsilon }|}\int _{\omega _{\varepsilon }} \nabla u_0(x)\cdot \nabla \varphi _v(x) \;dx, \end{aligned}$$

for all $v\in L^p({\textsf{D}})$. Subtracting the limit equation for $U_0$ yields

$$\begin{aligned}&\int _{\textsf{D}}(U_{\varepsilon }-U_0) v \;dx \\&\quad = \textrm{sgn}_{\Omega }(\{x_0\}) (\beta _2-\beta _1)\frac{1}{|\omega _{\varepsilon }|}\int _{\omega _{\varepsilon }} (\nabla K_{\varepsilon }\circ T_{\varepsilon }^{-1} - \nabla K\circ T_{\varepsilon }^{-1})\cdot \nabla \varphi _v(x) \;dx\\&\qquad + \textrm{sgn}_{\Omega }(\{x_0\}) (\beta _2-\beta _1)\frac{1}{|\omega _{\varepsilon }|}\int _{\omega _{\varepsilon }} \nabla K\circ T_{\varepsilon }^{-1} \cdot (\nabla \varphi _v(x)-\nabla \varphi _v(x_0)) \;dx\\&\qquad + \textrm{sgn}_{\Omega }(\{x_0\}) (\beta _2-\beta _1)\frac{1}{|\omega _{\varepsilon }|}\int _{\omega _{\varepsilon }} (\nabla u_0(x) - \nabla u_0(x_0))\cdot \nabla \varphi _v(x) \;dx \\&\qquad + \textrm{sgn}_{\Omega }(\{x_0\}) (\beta _2-\beta _1)\frac{1}{|\omega _{\varepsilon }|}\int _{\omega _{\varepsilon }} \nabla u_0(x)\cdot (\nabla \varphi _v(x)-\nabla \varphi _v(x_0)) \;dx \\&\quad =: I_1(v)+I_2(v)+I_3(v)+I_4(v). \end{aligned}$$

It is readily checked that using $\Vert \varphi _v\Vert _{C^1(\Omega \cup {\textsf{D}}{\setminus }{\overline{\Omega }})}\le \Vert v\Vert _{L_p({\textsf{D}})}$:

167 $$\begin{aligned} |I_1(v)|\le C\Vert \nabla K_{\varepsilon }-\nabla K\Vert _{L_1(\omega )^d}\Vert \nabla \varphi _v\Vert _{C^0(\overline{\omega _{\varepsilon }})^d} \le C\Vert \nabla K_{\varepsilon }-\nabla K\Vert _{L_1(\omega )^d}\Vert v\Vert _{L^p({\textsf{D}})} \end{aligned}$$

and

168 $$\begin{aligned} |I_3(v)| \le C|\omega _{\varepsilon }|^{-1}\Vert \nabla u_0-\nabla u_0(x_0)\Vert _{L^1(\omega _{\varepsilon })^d}\Vert v\Vert _{L^p({\textsf{D}})}. \end{aligned}$$

Now we recall the following equation which follows from the proof of Morrey’s inequality [35, p.280, Theorem 4]: for all $\varphi \in W^{2,p}(\Omega \cup {\textsf{D}}\setminus {\overline{\Omega }})$, $p>d$:

169 $$\begin{aligned} \frac{1}{|B_{\varepsilon }(x_0)|}\int _{B_{\varepsilon }(x_0)}|\nabla \varphi (x)-\nabla \varphi (x_0)|\;dx \le C\int _{B_{\varepsilon }(x_0)}\frac{|\nabla ^2\varphi (x)|}{|x-x_0|^{d-1}}\;dx. \end{aligned}$$

Now there are constants $\varrho >1$ and $C>0$, such that $|B_{{\varepsilon }\varrho }(x_0)|\le C|\omega _{{\varepsilon }}(x_0)|$ and $\omega _{\varepsilon }(x_0)\subset B_{{\varepsilon }\varrho }(x_0)$ for ${\varepsilon }>0$. Therefore,

170 $$\begin{aligned} \frac{1}{|\omega _{\varepsilon }(x_0)|}\int _{\omega _{\varepsilon }(x_0)}|\nabla \varphi (x)-\nabla \varphi (x_0)|\;dx \le C \frac{1}{|B_{{\varepsilon }\varrho }(x_0)|}\int _{B_{{\varepsilon }\varrho }(x_0)}|\nabla \varphi (x)-\nabla \varphi (x_0)|\;dx. \end{aligned}$$

Therefore it follows from Hölder’s inequality:

171 $$\begin{aligned} |I_2(v)|&\le C\Vert \nabla K\Vert _{C^0({\overline{\omega }})^d}\int _{B_{{\varepsilon }\varrho }(x_0)}\frac{|\nabla ^2\varphi _v(x)|}{|x-x_0|^{d-1}}\;dx \end{aligned}$$

172 $$\begin{aligned}&\le C\Vert \nabla K\Vert _{C^0({\overline{\omega }})^d}\Vert v\Vert _{L^p({\textsf{D}})} \left( \int _{B_{{\varepsilon }\varrho }(x_0)}\frac{1}{|x-x_0|^{p'(d-1)}}\;dx\right) ^{1/p'}. \end{aligned}$$

Changing variables yields

173 $$\begin{aligned} \left( \int _{B_{{\varepsilon }\varrho }(x_0)}\frac{1}{|x-x_0|^{p'(d-1)}}\;dx\right) ^{1/p'} = (\varrho {\varepsilon })^{\frac{d-p'(d-1)}{p'}}\left( \int _{B_1(0)}\frac{1}{|x|^{p'(d-1)}}\;dx\right) ^{1/p'} \end{aligned}$$

and the last integral is finite if $p'=q\in (1,\frac{d}{d-1})$, which is satisfied since $p>d$. Similarly we can show that

174 $$\begin{aligned} |I_4(v)|\le C{\varepsilon }^{\frac{d-p'(d-1)}{p'}}\Vert v\Vert _{L^p({\textsf{D}})}. \end{aligned}$$

Summarising we have shown that (recall $p'=q$)

175 $$\begin{aligned} \Vert U_{\varepsilon }-U_0\Vert _{L^q({\textsf{D}})}\le C({\varepsilon }^{\frac{d-q(d-1)}{q}} + \Vert \nabla K_{\varepsilon }-\nabla K\Vert _{L_1(\omega )^d} + |\omega _{\varepsilon }|^{-1}\Vert \nabla u_0-\nabla u_0(x_0)\Vert _{L^1(\omega _{\varepsilon })^d}) \end{aligned}$$

and the in view of right differentiability of $\nabla u_0$ near $x_0$, $|\omega _{\varepsilon }|^{-1}\Vert \nabla u_0-\nabla u_0(x_0)\Vert _{L^1(\omega _{\varepsilon })^d}\le C{\varepsilon }$ and the estimate (85). Moreover, the right hand side goes to zero as ${\varepsilon }\searrow 0$ in view of Lemma 3.5. $\square$

### Proof of Theorem 3.6

#### Proof of Theorem 3.6

We first note that multiplying (97) with ${\varepsilon }$ gives

176 $$\begin{aligned} \Vert u_{\varepsilon }- u_0 - {\varepsilon }K\circ T_{\varepsilon }^{-1} - {\varepsilon }^dv\Vert _{W^{1,q}({\textsf{D}})} \le C{\varepsilon }^{1+\frac{d}{q}}, \end{aligned}$$

and this yields by division by $|\omega _{\varepsilon }|$ with the definition $u_{\varepsilon }:= u_{\Omega _{\varepsilon }(x_0,\omega )}$ and $U_{\varepsilon }= \frac{u_{\varepsilon }-u_0}{|\omega _{\varepsilon }|}$ the estimate

177 $$\begin{aligned} \Vert U_{\varepsilon }- {\varepsilon }|\omega _{\varepsilon }|^{-1}K\circ T_{\varepsilon }^{-1} - |\omega |^{-1}v\Vert _{W^{1,q}({\textsf{D}})} \le C{\varepsilon }^{1+\frac{d}{q}-d}, \end{aligned}$$

where we note that the exponent $1+\frac{d}{q}-d>0$ for $q\in (1,\frac{d}{d-1})$. Now we estimate

178 $$\begin{aligned} \begin{aligned} \Vert U_{\varepsilon }- |\omega |^{-1}(R + v) \Vert _{L^q({\textsf{D}})} \le&\Vert U_{\varepsilon }- {\varepsilon }|\omega _{\varepsilon }|^{-1}K\circ T_{\varepsilon }^{-1} - |\omega |^{-1}v\Vert _{L^q({\textsf{D}})} \\&+ \Vert {\varepsilon }|\omega _{\varepsilon }|^{-1}K\circ T_{\varepsilon }^{-1} - |\omega |^{-1}R\Vert _{L^q({\textsf{D}})}. \end{aligned} \end{aligned}$$

The first term on the right hand side is bounded in view of (177). To treat the second term on the right hand side of (178), we change variables, recall ${\textsf{D}}_{\varepsilon }= T_{\varepsilon }^{-1}({\textsf{D}})$, and $R\circ T_{\varepsilon }= {\varepsilon }^{-(d-1)} R$ to obtain

179 $$\begin{aligned} \Vert {\varepsilon }|\omega _{\varepsilon }|^{-1}K\circ T_{\varepsilon }^{-1} - |\omega |^{-1}R\Vert _{L^q({\textsf{D}})}^q = {\varepsilon }^{d-q(d-1)} |\omega |^{-q}\Vert K- R\Vert _{L^q({\textsf{D}}_{\varepsilon })}^q. \end{aligned}$$

Now we recall $R(x) = \frac{Ax}{|x|^d}, K \in L^q(\omega )$ for $q\in [1,\frac{d}{d-1})$. In addition, since $K(x)-R(x)$ behaves as $|x|^{-d}$ for $|x|\rightarrow \infty$, we also have (similarly to Lemma 6.1) that $\Vert K-R\Vert _{L^q({\textbf{R}}^d\setminus \omega )}$ is bounded. It follows that

180 $$\begin{aligned} \Vert {\varepsilon }|\omega _{\varepsilon }|^{-1}K\circ T_{\varepsilon }^{-1} - |\omega |^{-1}R\Vert _{L^q({\textsf{D}})}^q \le {\varepsilon }^{d-q(d-1)} |\omega |^{-q}\Vert K- R\Vert _{L^q({\textbf{R}}^d)}^q. \end{aligned}$$

Finally noting that $\frac{d-q(d-1)}{q}<1$ is equivalent to $q>1$ this finishes the proof. $\square$

## Appendix

### Proof of Lemma 2.9

We start with $d=2$ and aim to derive an equation for $V_{\varepsilon }:=K_{\varepsilon }- K - v\circ T_{\varepsilon }-\ln ({\varepsilon })b$. Subtracting the weak formulation from the perturbed state equation (32) for ${\varepsilon }>0$ and ${\varepsilon }=0$ yields

$$\begin{aligned} \int _{\textsf{D}}\nabla (u_{\varepsilon }-u_0)\cdot \nabla \varphi \;dx=\int _{\omega _{\varepsilon }}(f_1-f_2)\varphi \;dx,\quad \text { for all }\varphi \in H^1_0({\textsf{D}}). \end{aligned}$$

Now the change of variables $T_{\varepsilon }(x)=y$, ${\textsf{D}}_{\varepsilon }=T_{\varepsilon }^{-1}({\textsf{D}})$, and dividing by ${\varepsilon }^2$ shows

$$\begin{aligned} \int _{{\textsf{D}}_{\varepsilon }}\nabla K_{\varepsilon }\cdot \nabla \varphi \;dx=\int _{\omega }(f_1-f_2)\varphi \;dx,\quad \text { for all }\varphi \in H^1_0({\textsf{D}}_{\varepsilon }). \end{aligned}$$

Note that, by extending $\varphi \in H^1_0({\textsf{D}}_{\varepsilon })$ by 0 in the exterior, we get

$$\begin{aligned} \int _{{\textsf{D}}_{\varepsilon }}\nabla K\cdot \nabla \varphi \;dx=\int _{\omega }(f_1-f_2)\varphi \;dx,\quad \text { for all }\varphi \in H^1_0({\textsf{D}}_{\varepsilon }). \end{aligned}$$

Hence, we conclude

$$\begin{aligned} \int _{{\textsf{D}}_{\varepsilon }}\nabla V_{\varepsilon }\cdot \nabla \varphi \;dx=0 ,\quad \text { for all }\varphi \in H^1_0({\textsf{D}}_{\varepsilon }), \end{aligned}$$

where we used that *v* is harmonic. Furthermore, we see that for $x\in \partial {\textsf{D}}_{\varepsilon }$ we have

$$\begin{aligned} V_{\varepsilon }(x)=-K(x)-v\circ T_{\varepsilon }(x)-\ln ({\varepsilon }) b=-K(x)+R({\varepsilon }x)-\ln ({\varepsilon })b=R(x)-K(x), \end{aligned}$$

where we used (39) and $b\in {\textbf{R}}$ is chosen such that $R({\varepsilon })=b\ln ({\varepsilon })$. Thus we observe, for ${\varepsilon }>0$ sufficiently small, there holds

$$\begin{aligned} |V_{\varepsilon }(x)|\le c|x|^{-1}+O(|x|^{-2}),\quad \text { for all }x\in \partial {\textsf{D}}_{\varepsilon }. \end{aligned}$$

Finally, we can apply [11, Lemma 3.4, Lemma 3.7] to conclude

$$\begin{aligned} \Vert V_{\varepsilon }\Vert _{\varepsilon }\le C \left( {\varepsilon }^{\frac{1}{2}}\Vert V_{\varepsilon }\Vert _{L^2(\partial {\textsf{D}}_{\varepsilon })}+\Vert V_{\varepsilon }\Vert _{H^{\frac{1}{2}}(\partial {\textsf{D}}_{\varepsilon })}\right) \le C{\varepsilon }. \end{aligned}$$

The proof for dimension $d\ge 3$ is similar. An identical computation shows that $V_{\varepsilon }:=K_{\varepsilon }- K - {\varepsilon }^{d-2}v\circ T_{\varepsilon }$ satisfies

$$\begin{aligned} \int _{{\textsf{D}}_{\varepsilon }}\nabla V_{\varepsilon }\cdot \nabla \varphi \;dx=0 ,\quad \text { for all }\varphi \in H^1_0({\textsf{D}}_{\varepsilon }), \end{aligned}$$

and one readily checks that for a.e. $x\in \partial {\textsf{D}}_{\varepsilon }$ there holds

$$\begin{aligned} V_{\varepsilon }(x)=-K(x)-{\varepsilon }^{d-2}v\circ T_{\varepsilon }(x)=-K(x)+{\varepsilon }^{d-2}R({\varepsilon }x)=R(x)-K(x), \end{aligned}$$

where in the last equality we used that *R* is homogenous of degree $-(d-2)$; see (40). Thus, an application of [11, Lemma 3.4, Lemma 3.7] yield

$$\begin{aligned} \Vert V_{\varepsilon }\Vert _{\varepsilon }\le C \left( {\varepsilon }^{\frac{1}{2}}\Vert V_{\varepsilon }\Vert _{L^2(\partial {\textsf{D}}_{\varepsilon })}+\Vert V_{\varepsilon }\Vert _{H^{\frac{1}{2}}(\partial {\textsf{D}}_{\varepsilon })}\right) \le C{\varepsilon }^{\frac{d}{2}}. \end{aligned}$$

$$\square $$

### Proof of Lemma 3.5

First note that, testing with $\varphi \in H^1_0({\textsf{D}}_{\varepsilon })$, we can rewrite (93) as

181 $$\begin{aligned} \int _{{\textsf{D}}_{\varepsilon }} \beta _{\omega \cup T_{\varepsilon }^{-1}(\Omega )}\nabla K\cdot \nabla \varphi \;dx =&\textrm{sgn}_{\Omega }(\{x_0\})(\beta _2-\beta _1)\int _\omega \nabla u_0(x_0)\cdot \nabla \varphi \;dx \end{aligned}$$

182 $$\begin{aligned}&+\textrm{sgn}_{\Omega }(\{x_0\})(\beta _2-\beta _1)\int _{T_{\varepsilon }^{-1}(\partial \Omega )}\partial _\nu K\varphi \;dS. \end{aligned}$$

Hence, combined with the rescaled equations (75) and (95), we see that $V_{\varepsilon }:=K_{\varepsilon }-K-{\varepsilon }^{d-1}v\circ T_{\varepsilon }$ satisfies

183 $$\begin{aligned}&\int _{{\textsf{D}}_{\varepsilon }}\beta _{\omega \cup T_{\varepsilon }^{-1}(\Omega )} \nabla V_{\varepsilon }\cdot \nabla \varphi \;dx\end{aligned}$$

184 $$\begin{aligned}&\quad =\textrm{sgn}_{\Omega }(\{x_0\})(\beta _2-\beta _1)\int _\omega \left( \nabla u_0\circ T_{\varepsilon }-\nabla u_0(x_0)\right) \cdot \nabla \varphi \;dx\end{aligned}$$

185 $$\begin{aligned}&\quad +\textrm{sgn}_{\Omega }(\{x_0\})(\beta _2-\beta _1)\int _{T_{\varepsilon }^{-1}(\partial \Omega )}\partial _\nu (K-R)\varphi \;dS=:F_{\varepsilon }(\varphi ). \end{aligned}$$

and $V_{\varepsilon }=K-R=:g_{\varepsilon }$ on $\partial {\textsf{D}}_{\varepsilon }$. From [11, Lemma 3.7] (in an $L^p$ setting) we deduce

186 $$\begin{aligned} \Vert V_{\varepsilon }\Vert _{L^q({\textsf{D}}_{\varepsilon })}+\Vert \nabla V_{\varepsilon }\Vert _{L^q({\textsf{D}}_{\varepsilon })^d}\le C\left( \Vert F_{\varepsilon }\Vert _{L^q({\textsf{D}}_{\varepsilon })}+{\varepsilon }^{1-\frac{1}{q}}\Vert g_{\varepsilon }\Vert _{L^q(\partial {\textsf{D}}_{\varepsilon })}+|g_{\varepsilon }|_{W^{1-\frac{1}{q},q}(\partial {\textsf{D}}_{\varepsilon })}\right) . \end{aligned}$$

In view of $|K(x)-R(x)|\approx |x|^{-d}$ for $|x|\rightarrow \infty$ and the scaling properties of Lemma 6.3 and Lemma 6.2, the result follows. $\square$

### Lemma 6.1

Let $\omega \subset {\textbf{R}}^d$ such that $0\in \omega$. Given a function $E:{\textbf{R}}^d\rightarrow {\textbf{R}}$ let $K(x):=\int _\omega E(x-y)\;dy$, for $x\in {\textbf{R}}^d$. Then we have the following properties: For dimension $d=2$ and $E(x):=\ln (|x|)$ there holds

(i) $$\Vert K-|\omega |E\Vert _{L^p({\textbf{R}}^2)}<\infty $$ for $$p\in (2,\infty )$$.
(ii) $$\Vert \nabla (K-|\omega |E)\Vert _{L^p({\textbf{R}}^2)^2}<\infty $$ for $$p\in (1,2)$$.

For dimension $d>2$ and $E(x):=|x|^{-k}$, $k\in {\textbf{N}}$ there holds

(iii) $$\Vert K-|\omega |E\Vert _{L^p({\textbf{R}}^d)}<\infty $$ for $$p\in (\frac{d}{k+1},\frac{d}{k})$$.
(iv) $$\Vert \nabla (K-|\omega |E)\Vert _{L^p({\textbf{R}}^d)^d}<\infty $$ for $$p\in (\frac{d}{k+2},\frac{d}{k+1})$$.

### Proof

We start with the two dimensional case. Hence, let $E(x)=\ln (|x|)$. Note that a Taylor expansion shows that there is a $R>0$

187 $$\begin{aligned} K(x)-|\omega |E(x)=\int _\omega E(x-y)-E(x)\;dx\le C|x|^{-1},\quad \text { for }|x|>R. \end{aligned}$$

Now splitting the integral with respect to this constant *R* we get for all $p\in [1,\infty )$:

188 $$\begin{aligned} \Vert K-|\omega |E\Vert ^p_{L^p(B_R(0))}\le \Vert K\Vert ^p_{L^p(B_R(0))}+\Vert |\omega |E\Vert ^p_{L^p(B_R(0))}<\infty \end{aligned}$$

since $\ln (|x|)\in L_p^{\text {loc}}({\textbf{R}}^2)$ and the same holds for *K*. For the second part we use (187) to conclude for all $p\in (2,\infty )$:

189 $$\begin{aligned} \Vert K-|\omega |E\Vert ^p_{L^p(B_R(0)^c)}\le C\int _{B_R(0)^c}|x|^{-p}\;dx=C\int _R^\infty r^{1-p}\;dr<\infty , \end{aligned}$$

which shows (i). From (187) we further see that

190 $$\begin{aligned} \nabla \left( K(x)-|\omega |E(x)\right) \le C|x|^{-2},\quad \text { for }|x|>R. \end{aligned}$$

Now, noting that $|\nabla E(x)|=|x|^{-1}$, we see that for all $p\in (1,2)$:

191 $$\begin{aligned} \Vert \nabla E\Vert ^p_{L^p(B_R(0))^2}=\int _0^Rr^{1-p}\;dr<\infty . \end{aligned}$$

Since the same holds true for *K*, we conclude $\Vert \nabla (K-|\omega |E)\Vert _{L^p(B_R(0))^2}<\infty$ for all $p\in (1,2)$. For the exterior domain, we again use (187) to conclude for all $p\in (1,\infty )$:

192 $$\begin{aligned} \Vert \nabla \left( K-|\omega |E\right) \Vert ^p_{L^p(B_R(0)^c)^2}\le C\int _{B_R(0)^c}|x|^{-2p}\;dx=C\int _R^\infty r^{1-2p}\;dr<\infty . \end{aligned}$$

Combining (191) and (191) yields (ii). Similar arguments, exploiting the Taylor expansion of $|x|^{-k}$ for $k\in {\textbf{N}}$, shows item (iii) and (iv). $\square$

### Lemma 6.2

For $x_0\in {\textsf{D}}$ and ${\varepsilon }>0$ let $T_{\varepsilon }(x):=x_0+{\varepsilon }x$ and ${\textsf{D}}_{\varepsilon }:=T_{\varepsilon }^{-1}({\textsf{D}})$. Further define for $1<p<\infty$ the scaled norm

193 $$\begin{aligned} \Vert \varphi \Vert _{{\varepsilon },p}:={\varepsilon }\Vert \varphi \Vert _{L_p({\textsf{D}}_{\varepsilon })}+\Vert \nabla \varphi \Vert _{L_p({\textsf{D}}_{\varepsilon })^d},\quad \text { for all }\varphi \in W^{1,p}({\textsf{D}}_{\varepsilon }). \end{aligned}$$

Then there holds:

(i) $$\Vert \varphi \circ T_{\varepsilon }^{-1}\Vert _{W^{1,p}({\textsf{D}})}={\varepsilon }^{\frac{d}{p}-1}\Vert \varphi \Vert _{{\varepsilon },p}$$.
(ii) $$\Vert \varphi \circ T_{\varepsilon }^{-1}\Vert _{L_p(\partial {\textsf{D}})}={\varepsilon }^{\frac{d-1}{p}}\Vert \varphi \Vert _{L_p(\partial {\textsf{D}}_{\varepsilon })}$$.
(iii) $$|\varphi \circ T_{\varepsilon }^{-1}|_{W^{\alpha ,p}(\partial {\textsf{D}})}={\varepsilon }^{\frac{d-1}{p}-\alpha }|\varphi |_{W^{\alpha ,p}(\partial {\textsf{D}}_{\varepsilon })}$$.
(iv) $$\Vert \varphi \Vert _{{\varepsilon },p}\le C\left( {\varepsilon }^{1-\frac{1}{p}}\Vert \varphi \Vert _{L_p(\partial {\textsf{D}}_{\varepsilon })}+|\varphi |_{W^{1-\frac{1}{p},p}(\partial {\textsf{D}}_{\varepsilon })}\right) $$.

### Proof

ad (i): This is a direct consequence of the scaling of $L_p$ norms.ad (ii): The same argument as before, considering $\text {dim}(\partial {\textsf{D}})=d-1$.ad (iii): We have

$$\begin{aligned} |\varphi \circ T_{\varepsilon }^{-1}|_{W^{\alpha ,p}(\partial {\textsf{D}})}^p=&\int _{\partial {\textsf{D}}}\int _{\partial {\textsf{D}}}\frac{|\varphi \circ T_{\varepsilon }^{-1}(x)-\varphi \circ T_{\varepsilon }^{-1}(y)|^p}{|x-y|^{\alpha p+d-1}}\;dxdy\\ =&{\varepsilon }^{2d-2}\int _{\partial {\textsf{D}}_{\varepsilon }}\int _{\partial {\textsf{D}}_{\varepsilon }}\frac{|\varphi (x)-\varphi (y)|^p}{|(x_0+{\varepsilon }x)-(x_0+{\varepsilon }y)|^{\alpha p+d-1}}\;dxdy\\ =&{\varepsilon }^{d-1-\alpha p}\int _{\partial {\textsf{D}}_{\varepsilon }}\int _{\partial {\textsf{D}}_{\varepsilon }}\frac{|\varphi (x)-\varphi (y)|^p}{|x-y|^{\alpha p+d-1}}\;dxdy\\ =&{\varepsilon }^{d-1-\alpha p}|\varphi |_{W^{\alpha ,p}(\partial {\textsf{D}})}^p. \end{aligned}$$

ad (iv): Using the previous scalings and the right-inverse extension operator on ${\textsf{D}}$, we get

$$\begin{aligned} \Vert \varphi \Vert _{{\varepsilon },p}=&{\varepsilon }^{1-\frac{d}{p}}\Vert \varphi \circ T_{\varepsilon }^{-1}\Vert _{W^{1,p}({\textsf{D}})}\\ \le&C{\varepsilon }^{1-\frac{d}{p}}\left( \Vert \varphi \circ T_{\varepsilon }^{-1}\Vert _{L_p(\partial {\textsf{D}})}+|\varphi \circ T_{\varepsilon }^{-1}|_{W^{1-\frac{1}{p},p}(\partial {\textsf{D}})}\right) \\ =&C{\varepsilon }^{1-\frac{d}{p}}\left( {\varepsilon }^{\frac{d-1}{p}}\Vert \varphi \Vert _{L_p(\partial {\textsf{D}}_{\varepsilon })}+{\varepsilon }^{\frac{d-1}{p}-(1-\frac{1}{p})}|\varphi |_{W^{1-\frac{1}{p},p}(\partial {\textsf{D}}_{\varepsilon })}\right) \\ =&C\left( {\varepsilon }^{1-\frac{1}{p}}\Vert \varphi \Vert _{L_p(\partial {\textsf{D}}_{\varepsilon })}+|\varphi |_{W^{1-\frac{1}{p},p}(\partial {\textsf{D}}_{\varepsilon })}\right) . \end{aligned}$$

$$\square $$

### Lemma 6.3

Let $g(x):=|x|^{-d}$, for $x\in {\textbf{R}}^d$. Then there holds

(i) $$\Vert g\Vert _{L_p(\partial {\textsf{D}}_{\varepsilon })}\le C{\varepsilon }^{\frac{1-d}{p}+d}$$.
(ii) $$|g|_{W^{\alpha ,p}(\partial {\textsf{D}}_{\varepsilon })}\le C{\varepsilon }^{\frac{1-d}{p}+d+\alpha }$$.

### Proof

ad (i): We have

194 $$\begin{aligned} \Vert g\Vert _{L_p(\partial {\textsf{D}}_{\varepsilon })}^p=\int _{\partial {\textsf{D}}_{\varepsilon }}|x|^{-dp}\;dx={\varepsilon }^{1-d}\int _{\partial {\textsf{D}}}\left| \frac{x-x_0}{{\varepsilon }}\right| ^{-dp}\;dx\le C{\varepsilon }^{1-d+dp}. \end{aligned}$$

ad (ii): Similarly we conclude

$$\begin{aligned} |g|_{W^{\alpha ,p}(\partial {\textsf{D}}_{\varepsilon })}^p=&\int _{\partial {\textsf{D}}_{\varepsilon }}\int _{\partial {\textsf{D}}_{\varepsilon }}\frac{|g(x)-g(y)|^p}{|x-y|^{p\alpha +d-1}}\\ =&{\varepsilon }^{2-2d}\int _{\partial {\textsf{D}}}\int _{\partial {\textsf{D}}}\frac{|g\circ T_{\varepsilon }^{-1}(x)-g\circ T_{\varepsilon }^{-1}(y)|^p}{|x-y|^{p\alpha +d-1}{\varepsilon }^{-(p\alpha +d-1)}}\\ =&{\varepsilon }^{1-d+p\alpha }\int _{\partial {\textsf{D}}}\int _{\partial {\textsf{D}}}\frac{|g\circ T_{\varepsilon }^{-1}(x)-g\circ T_{\varepsilon }^{-1}(y)|^p}{|x-y|^{p\alpha +d-1}}\\ \le&C{\varepsilon }^{1-d+p\alpha }{\varepsilon }^{-p+p(d+1)}\\ =&C{\varepsilon }^{1-d+p(d+\alpha )}, \end{aligned}$$

where we used $|g\circ T_{\varepsilon }^{-1}(x)-g\circ T_{\varepsilon }^{-1}(y)|\approx {\varepsilon }^{-1}|\nabla (g\circ T_{\varepsilon }^{-1})(x)\cdot (x-y)|$ and $\nabla g(x)\approx |x|^{-(d+1)}$. For a more detailed proof of this estimate we refer to [11, Lemma 4.4]. $\square$

## Footnotes

1 The Beppo-Space $\dot{\text {BL}}({\textbf{R}}^d)$ is defined as the quotient space $\{\varphi \in H^1_{loc}({\textbf{R}}^d): \nabla \varphi \in L^2({\textbf{R}}^d)^d\}/{\textbf{R}}$, where $/{\textbf{R}}$ means we quotient out constants. This space is equipped with the norm $\Vert \varphi \Vert _{\dot{\text {BL}}({\textbf{R}}^d)}:= \Vert \nabla \varphi \Vert _{L^2({\textbf{R}}^d)^d}$; see [9, 33].
